# Quantitative susceptibility mapping in myotonic dystrophy: clinical relevance of subcortical iron accumulation

**DOI:** 10.1093/braincomms/fcag017

**Published:** 2026-01-20

**Authors:** Cristiana Fiscone, Magali J Rochat, Silvia De Pasqua, Micaela Mitolo, Gianfranco Vornetti, Fiorina Bartiromo, Lorenzo Cirignotta, Fabio Pizza, Marianna Nardozza, Greta Venturi, David Neil Manners, Patrizia Avoni, Rocco Liguori, Caterina Tonon, Raffaele Lodi

**Affiliations:** Department of Biomedical and Neuromotor Sciences, University of Bologna, Bologna 40126, Italy; Functional and Molecular Neuroimaging Unit, IRCCS Istituto delle Scienze Neurologiche di Bologna, Bologna 40139, Italy; UOC Clinica Neurologica, IRCCS Istituto delle Scienze Neurologiche di Bologna, Bologna 40139, Italy; Functional and Molecular Neuroimaging Unit, IRCCS Istituto delle Scienze Neurologiche di Bologna, Bologna 40139, Italy; Department of Medicine and Surgery, University of Parma, Parma 43125, Italy; Department of Biomedical and Neuromotor Sciences, University of Bologna, Bologna 40126, Italy; Functional and Molecular Neuroimaging Unit, IRCCS Istituto delle Scienze Neurologiche di Bologna, Bologna 40139, Italy; Functional and Molecular Neuroimaging Unit, IRCCS Istituto delle Scienze Neurologiche di Bologna, Bologna 40139, Italy; Department of Experimental, Diagnostic and Specialty Medicine, University of Bologna, Bologna 40138, Italy; Department of Biomedical and Neuromotor Sciences, University of Bologna, Bologna 40126, Italy; UOC Clinica Neurologica, IRCCS Istituto delle Scienze Neurologiche di Bologna, Bologna 40139, Italy; Cardiology Department, Bellaria Hospital, Bologna 40139, Italy; Functional and Molecular Neuroimaging Unit, IRCCS Istituto delle Scienze Neurologiche di Bologna, Bologna 40139, Italy; Functional and Molecular Neuroimaging Unit, IRCCS Istituto delle Scienze Neurologiche di Bologna, Bologna 40139, Italy; Department for Life Quality Sciences, University of Bologna, Rimini 47921, Italy; Department of Biomedical and Neuromotor Sciences, University of Bologna, Bologna 40126, Italy; UOC Clinica Neurologica, IRCCS Istituto delle Scienze Neurologiche di Bologna, Bologna 40139, Italy; Department of Biomedical and Neuromotor Sciences, University of Bologna, Bologna 40126, Italy; UOC Clinica Neurologica, IRCCS Istituto delle Scienze Neurologiche di Bologna, Bologna 40139, Italy; Department of Biomedical and Neuromotor Sciences, University of Bologna, Bologna 40126, Italy; Functional and Molecular Neuroimaging Unit, IRCCS Istituto delle Scienze Neurologiche di Bologna, Bologna 40139, Italy; Department of Biomedical and Neuromotor Sciences, University of Bologna, Bologna 40126, Italy; Functional and Molecular Neuroimaging Unit, IRCCS Istituto delle Scienze Neurologiche di Bologna, Bologna 40139, Italy

**Keywords:** quantitative susceptibility mapping, myotonic dystrophy type 1, subcortical structures, biomarker, disease progression

## Abstract

Myotonic dystrophy type 1 is a dominantly inherited disorder, affecting musculoskeletal and central nervous systems and mainly characterized by progressive muscular atrophy and multisystemic damages including cardiac, respiratory and sleep dysfunctions. Neuroimaging studies conducted in myotonic dystrophy type 1 patients have documented widespread cerebral alterations encompassing structural, microstructural, functional and metabolic aspects of the brain, while comparatively few studies have investigated the role of iron concentration in the pathophysiology of central nervous system impairment. We report here the use of quantitative susceptibility (χ) mapping to explore iron content of both cortical and subcortical structures in myotonic dystrophy type 1 patients and to assess its possible clinical relevance, combining imaging and clinical data. Thirty-four myotonic dystrophy type 1 participants (20 females, 46.8 ± 12.0 years old) and 35 age- and sex- matched healthy controls (20 females, 50.5 ± 17.3 years old) were included in the study. All participants underwent MRI examinations in the same 3-Tesla scanner. The MRI protocol included 3D morphological T_1_-weighted magnetization prepared rapid gradient echo and T_2_*weighted multi-echo gradient echo for quantitative susceptibility mapping reconstruction. Cortical and subcortical structures were automatically segmented, and a volume of interest–based analysis was performed; χ distributions were compared between the two groups and myotonic dystrophy type 1 χ values were correlated with clinical and laboratory data. In the myotonic dystrophy type 1 group, a significant increase of χ was found in almost all cortical gyri, as a non-specific sign of neurodegeneration. Among subcortical structures, χ was significantly higher in myotonic dystrophy type 1 group in both thalamus (ventral and pulvinar nuclei) and brainstem (pons and medulla), compared to healthy controls. Additionally, correlation analysis showed some links between χ in subcortical structures and clinical signs, suggesting greater iron concentration with deterioration of clinical conditions. Thalamic χ values were associated with cardiological parameters and disability scores and, as with brainstem χ, they were also positively correlated with the number of central apnoeas; finally, thalamic and brainstem χ were negatively correlated with the age of onset. This study showed a correlation between autonomic dysfunction related to certain subcortical structures and their χ; higher values of χ correlated with greater functional impairment, suggesting iron accumulation detected by the quantitative susceptibility mapping technique is a possible biomarker of disease progression.

## Teaser

Fiscone *et al*. used quantitative susceptibility mapping to measure iron in cortical and subcortical regions of patients with myotonic dystrophy type 1. They observed increased iron in the thalamus and brainstem, which correlated with clinical severity and autonomic dysfunction, suggesting iron accumulation as a potential biomarker of disease progression.

## Graphical Abstract

**Graphical Abstract:**
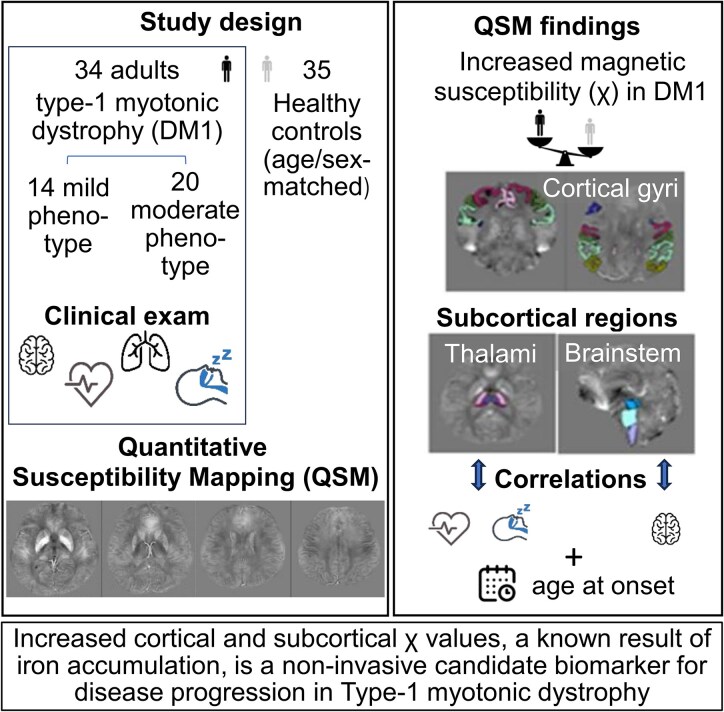

## Introduction

Myotonic dystrophy type 1 (DM1 or Steinert’s disease) is one of the most frequent forms of inherited muscular dystrophy in adults.^1^ This genetic disorder is caused by an unstable trinucleotide cytosine-thymine-guanine (CTG) repeat expansion in the DM protein kinase (DMPK) gene, leading to an accumulation of mutant RNA aggregates. The variability of genetic mutations demonstrates tissue-specific somatic mosaicism of increasing repeat length, resulting in a wide spectrum of clinical signs.

DM1 is a progressive multisystemic disorder entailing, among other characteristics, a progressive muscle involvement with myotonia and dystrophy, cardiac involvement, respiratory disturbances endocrine and gastrointestinal disorders.^2,3^ Moreover, a CNS dysfunction with sleep disorder, cognitive impairment, behavioural and psychiatric disorders is now widely recognized. Respiratory complications and cardiac arrhythmias are the most common cause of death in DM1; however, the central and/or peripherical contribution to the pathogenesis of respiratory decline and cardiac manifestation are still ill-defined.^4,5^

DM1 phenotypization is further complicated by the existence of a broad clinical spectrum of DM1. Classification into different subgroups can vary in terms of symptom severity (mild, classic and severe), age at onset of disease manifestations (congenital, infantile/juvenile, adult-onset and late-onset) and triplet repeat expansion (E1, E2 and E3, characterized by an increasing severity of neuromuscular and cognitive symptomatology).^3,6-8^ Although it is generally accepted that the number of the repeats correlates with age at onset and disease severity,^9^ the association between the CTG repeat size and some clinical features remains controversial due to somatic tissue mosaicism and the limited size of the cohorts assessed across studies.^10-12^

Advanced MRI studies have investigated CNS involvement in DM1^13^: they have showed a general atrophy and widespread grey matter (GM) volume reductions in all cortical lobes^14^ and deep structures,^15^ and a prevalence of subcortical and periventricular white matter (WM) hyperintensities,^16,17^ while normal appearing WM might conceal alterations in both MR spectroscopic^18^ and microstructural parameters;^19^ functional anomalies, such as abnormal resting-state networks, have also been reported.^20-22^

However, an imaging biomarker that monitors the spatiotemporal evolution of the disorder and its relevance for clinical symptoms is still needed.^23^ In the CNS, iron is involved in many vital processes such as metabolism, myelin synthesis and neurotransmitter production.^24^ Notably, changes in iron homoeostasis have been correlated to ageing while altered cellular iron distribution and accumulation have been reported in different neurodegenerative diseases.^25-27^ Excessive levels of brain iron are widely implicated in neuroinflammatory processes as part of the oxidative stress cascade and microglial activation.^28^

MRI sequences that are sensitive to tissue magnetic susceptibility are of great interest, as these permit *in vivo* evaluation of brain iron. Quantitative susceptibility mapping (QSM) is an advanced MRI technique that provides voxel-wise mapping of tissue magnetic susceptibility χ,^29,30^ facilitating the quantification of the local concentration of elements such as myelin and iron-containing molecules.^31^ In the past decade, QSM has been used to reveal distinct brain iron profiles in neurodegenerative diseases such as Alzheimer’s disease and Parkinson’s disease, correlated with disease duration and severity (see Ravanfar *et al*.^32^ for a systematic review). Surprisingly, to date, only one study has explored brain magnetic susceptibility in DM1: results highlighted greater atrophy and a broader magnetic susceptibility among subcortical GM nuclei in DM1 patients, while iron accumulation was significantly associated with different clinical symptoms.^33^

Despite the interesting data of this exploratory study, the limited cohort and heterogeneous presentation of patients prevented definite conclusions from being drawn regarding the pathogenesis of brain iron accumulation in DM. Here, we propose an original study, analysing multiple brain structures within a sample homogenous in terms of phenotypic abundance and well characterized clinically, to address the following questions: is there a distinct brain iron profile for DM1 patients compared to healthy controls and can the pattern of magnetic susceptibility also differentiate between genetic categories of DM1 patients? Are the changes in brain magnetic susceptibility associated with changes in brain volume or age of disease onset? Do the regional changes of magnetic susceptibility correlate with the clinical manifestations of Steinert’s disease? Briefly, we used QSM to explore brain tissue magnetic susceptibility and find more specific biomarkers to characterize the disease.

## Materials and methods

### Study sample

We enrolled 36 DM1 patients with no contraindications to MRI, recruited within a prospective study at the Neuromuscular Center within the UOC Neurological Clinic of the IRCCS Institute of Neurological Sciences, Bologna, IT, between May and November 2021. We decided to use the classification based on genetics since the one based on the onset age leads to disproportionate classes unsuitable for comparison [4 congenital/childhood (birth to 10 years old), 23 juvenile/adult (11–40 years old) and 9 late onset (>40 years old)]. CTG triplet expansion sizes were measured in all patients in genomic DNA extracted by peripheral blood leukocyte using Southern blot analysis. Depending on the number of repeat expansions, patients were classified into three genetic classes^34-36^: 14 patients E1 (50–150 CTG repeats), 20 patients E2 (150–1000 CTG repeats) and 2 patients E3 (>1000 CTG repeats). We did not consider E3 class patients because of the limited number and the poor quality of susceptibility images. A final sample of 34 DM1 patients was thus included in this study (F/M: 20/14; 46.8 ± 12.0 years).

The study protocol was approved by the local Ethical Committee (no. 1088–2020-OSS-AUSLBO). All DM1 patients underwent a standardized clinical (neurological, cardiological and respiratory) and neuroradiological evaluation.

A cohort of 35 sex- and age- matched healthy control subjects (HC) (F/M 20/15; 50.5 ± 17.4 years) were also included for comparisons; MRI data acquired using the same study protocol were obtained from the healthy controls database of Neuroimaging Laboratory (IRCCS Institute of Neurological Sciences of Bologna, Bellaria Hospital, IT). This database was purposely designed to collect normative values of quantitative MR parameters for clinical and research purposes. The anamnestic absence of neuropsychiatric disorders was verified for each HC. Clinical assessment was provided for DM1 patients only. Demographic characteristics of patients and controls are reported in Table 1.

**Table 1:** Main characteristics of DM1 patients (as a whole cohort and divided into E1 and E2 genetic classes) and HCs

	DM1	HC	*P*-value
*N*	34	35	
Sex, F:M	20:14	20:15	0.888
Age, years	46.8 ± 12.0 (20–71)	50.5 ± 17.4 (24–86)	0.230
Onset age, years	30.0 ± 15.7 (4–68)		
Disease duration, months	201.0 ± 116.8 (7–433)		

	E1 (50–150 CTG repeats)	E2 (150–1000 CTG repeats)	*P*-value
*N*	14	20	0.389
Sex, F:M	7:7	13:7	
Age, years	50.3 ± 10.0 (28–71)	44.3 ± 12.9 (22–67)	0.234
Onset age, years	36.4 ± 14.8 (16–68)	25.6 ± 15.0 (4–55)	0.044*
Disease duration, months	166.6 ± 110.2 (7–351)	225.2 ± 117.8 (76–433)	0.152

Demographic values are given as mean ± standard deviation (min–max) and compared between groups (*t*-test; *P < 0.05).

### Clinical evaluation

Neurological, pneumological and cardiological evaluations were performed on the patient cohort, together with sleep recordings study^37^ and MR scans (Section 2.3), allowing the combination of imaging and clinical data. Values of clinical data are reported in Table 2, for the whole DM1 sample and for E1 and E2 classes individually.

**Table 2:** Neurological, pneumological, cardiological and sleep recording data (mean ± standard deviation) of DM1 patients

Clinical evaluation	Clinical measurements	Pathological cut-offs	DM1	E1	E2	*P*-value
Neurological evaluation	MIRS		3.15 ± 0.78	2.79 ± 0.89	3.40 ± 0.60	0.022*
	NIFDS (NP)		3.34 ± 2.70	2.91 ± 2.30	3.61 ± 2.95	0.507
	NIFDS (Mo)		9.41 ± 4.01	6.82 ± 3.34	11.00 ± 3.60	0.004*
	NIFDS (My)		5.48 ± 2.71	3.91 ± 2.66	6.44 ± 2.31	0.012*
	NIFDS (DL)		2.10 ± 1.80	0.91 ± 0.94	2.83 ± 1.83	0.003**
	NIFDS (tot)		20.34 ± 8.80	14.55 ± 5.18	23.89 ± 8.75	0.003**
	ESS	≥10	8.25 ± 2.92 (Pat = 20.58%)	8.54 ± 2.93 (Pat = 21.43%)	8.05 ± 2.97 (Pat = 20.00%)	0.651
Pneumological evaluation	FVC	≤80	84.24 ± 17.80 (Pat = 64.70%)	90.29 ± 15.32 (Pat = 71.43%)	80.00 ± 18.53 (Pat = 60.00%)	0.098
	PaCO_2_	≥45	45.45 ± 4.96 (Pat = 52.94%)	43.86 ± 4.54 (Pat = 35.71%)	46.76 ± 5.01 (Pat = 65.00%)	0.122
	PaO_2_	≤80	85.98 ± 10.15 (Pat = 20.58%)	87.47 ± 10.63 (Pat = 21.43%)	84.92 ± 9.99 (Pat = 20.00%)	0.516
Cardiological evaluation	PR (ms)	≥200	194.54 ± 34.37 (Pat = 38.23%)	190.50 ± 41.20 (Pat = 57.14%)	197.80 ± 28.90 (Pat = 25.00%)	0.593
	QRS (ms)	≥120	116.25 ± 25.15 (Pat = 29.41%)	118.58 ± 28.16 (Pat = 14.28%)	120.00 ± 22.91 (Pat = 40.00%)	0.391
	EF	F: ≤54; M: ≤52	60.72 ± 6.39 (Pat = 88.23%)	58.57 ± 4.21 (Pat = 85.71%)	62.19 ± 7.27 (Pat = 90.00%)	0.117
Sleep recordings	ODI		11.46 ± 11.10	8.58 ± 6.80	13.76 ± 13.41	0.235
	OAHI	≥15	11.36 ± 10.49 (Pat = 23.52%)	8.80 ± 6.43 (Pat = 7.14%)	13.40 ± 12.73 (Pat = 35.00%)	0.266
	CAHI	≥15	1.26 ± 1.69 (Pat = 0%)	1.01 ± 1.57	1.46 ± 1.81	0.500

Values are given as mean ± standard deviation; for each clinical index, values in brackets report for each DM1, E1 and E2 group the proportion of patients with a score over the pathological cut-off (Pat = above pathological cut-off), significant (*P < 0.05) and highly significant (**P < 0.01) differences between E1 and E2 classes are highlighted. Neurological evaluation: MIRS, Muscular Impairment Rating Scale; NIFDS, Neuromuscular Impairment Function and Disability Scale; NP, neuropsychological; Mo, motor; My, myotonia; DL, daily life activity; tot, total score; ESS, Epworth Sleepiness Scale. Pneumological evaluation: FVC, forced vital capacity, is reported as % of the predicted value; PaCO_2_, arterial carbon dioxide partial pressure; PaO_2_, arterial oxygen partial pressure. Cardiological evaluation: EF, ejection fraction; F, females; M, males. Sleep recordings: ODI, oxygen desaturation index; OAHI, obstructive apnoea hypopnoea index; CAHI, central apnoeas hypopnoea index. OAHI and CAHI are reported as number of apnoeas, hypopnoeas and central apnoeas per hour of recording time. When available, pathological cut-offs have been indicated in the dedicated column.

#### Neurological examination

Each patient was enrolled in the study during a first neurological physical examination by expert neurologists. Neuromuscular impairment and disability were assessed according to two validated DM1-specific rating scales: (i) the Muscular Impairment Rating Scale (MIRS),^3^ a 5-point rating scale of 1 (no muscular impairment) to 5 (severe proximal weakness), developed to characterize the distal to proximal progression of muscular involvement, and (ii) the Neuromuscular Impairment Function and Disability Scale (NIFDS)^35^ consisting in 21 ordinal items divided into four domains: neuropsychological domain (NP, maximum score: 20), motor domain (MO, maximum score: 35), myotonia domain (MY, maximum score: 12) and daily life activity domain (DL maximum score: 15). The total score ranges from 0 (normal) to 82 (worst condition).

Additionally, the presence of excessive daytime sleepiness (EDS) was assessed by the Italian version of the Epworth Sleepiness Scale (ESS),^38^ with scores > 10 considered pathological.

#### Cardiological evaluation

Cardiological assessment included clinical evaluation, ECG, 24 h ECG Holter monitoring and trans-thoracic echocardiogram. Conduction abnormalities and arrhythmia on a standard ECG or 24 h ECG Holter including PR interval > 200 ms (first grade atrio-ventricular block), QRS duration > 120 ms, right or left bundle branch block and delayed intraventricular conduction, atrial fibrillation or flutter were considered indicative of cardiac involvement. We measured systolic left ventricular dysfunction as reduction of ejection fraction (EF) below 52% for men and below 54% for women.^39^

#### Pneumological evaluation

Arterial blood gas analysis was performed with recording of arterial oxygen partial pressure (PaO_2_) and arterial carbon dioxide partial pressure (PaCO_2_); we defined hypercapnia as partial carbon dioxide pressure PaCO_2_ > 45 mmHg and hypoxaemia as PaO_2_ < 80 mmHg.

Pulmonary function tests were used to assess forced vital capacity (FVC); we defined a restrictive defect when FVC was <80% of the predicted value.

#### Sleep recordings

In selected cases (27 out 34 patients) with respiratory disorders in sleep and/or excessive daytime sleepiness (EDS, assessed by the Epworth scale), a cardiorespiratory study was performed in order to verify the presence of a nocturnal respiratory disturbance. Phasic respiratory events were scored according to AASM guidelines:

- Oxygen desaturation index (ODI) was defined as the arithmetic mean of desaturations > 4% of baseline per hour of recording time.
- Obstructive apnoea hypopnoea index (OAHI) was defined as the arithmetic mean of the number of apnoeas or hypopnoeas per hour of recording time. An index of 5–14.9 was considered as mild, 15–30 as moderate and an index > 30 as severe.
- Central apnoeas hypopnoea index (CAHI) was defined by the absence of airflow and thoracoabdominal movements > 10 s.

### Magnetic resonance protocol and processing

Participants underwent a standardized brain MR acquisition protocol on a 3T clinical scanner (Magnetom Skyra; Siemens Healthineers, Erlangen, Germany), equipped with a whole-body transmit and a 64-channel Siemens Head/Neck receiver coil. The MR protocol provided T_1_w magnetization prepared rapid gradient echo (MPRAGE) (3D sagittal TR/TI/TE = 2300/900/2.98 ms, 1 × 1 × 1 mm^3^, FA 9°, scan duration ∼ 5 min) and T_2_*w gradient echo (3D axial, nTEs = 5, TR/TE/ΔTE = 53/9.42/9.42 ms, 0.5 × 0.5 × 1.5 mm^3^, FA 15°, scan duration ∼ 9 min). From the T_2_*w phase maps, susceptibility maps were obtained through Laplacian unwrapping,^40^ variable kernel sophisticated harmonic artifact reduction for phase data (V-SHARP) (Li, 2011) as background removal, weighted averaging of echo times^41^ and iterative least square (iLSQR)^42^ as dipole inversion—STI Suite.^4^ CSF was selected as a reference. The QSM processing protocol is described in detail in Fiscone *et al*.^4^

QSM volumes were linearly registered to the corresponding MPRAGE volume [Functional Magnetic Resonance Imaging of the Brain (FMRIB) Software Library (FSL) (v. 6.0.4)^43^—FSL’s Linear Image Registration Tool (FLIRT)].^44,45^ QSM-derived bulk susceptibility measures were used to provide a quantitative map of magnetic susceptibility, which reflects the concentration of both diamagnetic (calcium, myelin) and paramagnetic substances (iron) in the brain tissue.

### Magnetic resonance image segmentation

**Figure 1:**
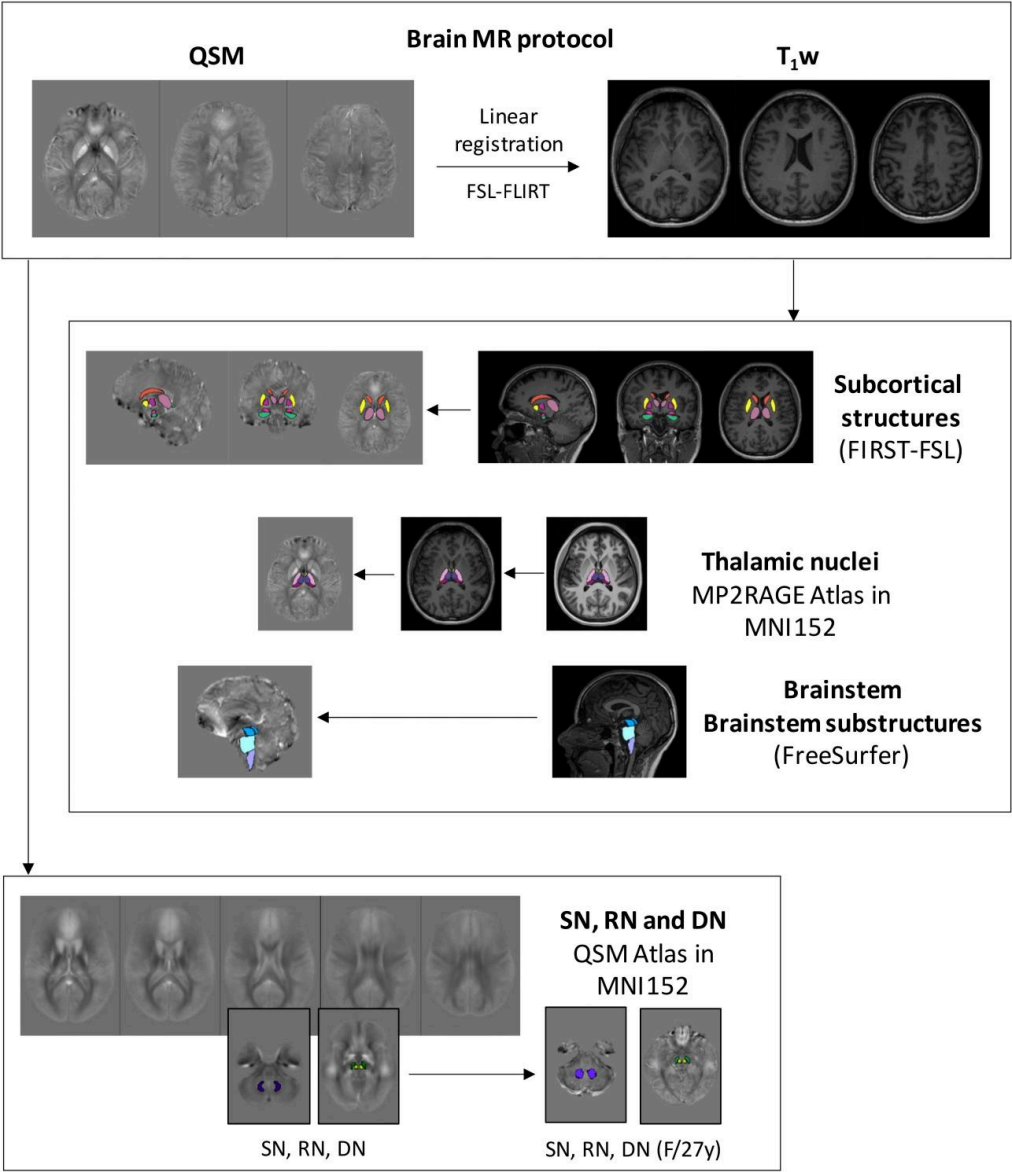
**Workflow pipeline, including MR protocol and segmentation methods.** Ten cortical gyri (precentral gyrus, caudal middle frontal gyrus, paracentral gyrus and pars-triangularis in frontal lobe, postcentral gyrus, inferior parietal gyrus and supramarginal gyrus in parietal lobe, transverse temporal gyrus in temporal lobe and posterior cingulate cortex and isthmus in the cingulate cortex) were selected from FreeSurfer cortical parcellations; the brainstem and its substructures (midbrain, pons and medulla) from a tool of Freesurfer^50^; caudate, accumbens, putamen, globus pallidus, thalamus, hippocampus and amygdala from FIRST; subthalamic nuclei (anterior, medial, ventral and pulvinar) from an MP2RAGE-enhanced atlas developed in Brun *et al*.^49^; substantia nigra (SN), red (RN) and dentate nuclei (DN) from a study-specific susceptibility-enhanced atlas. MR, magnetic resonance; T_1_w, T_1_-weight sequences; FIRST, FMRIB's Integrated Registration and Segmentation Tool; FSL, FMRIB Software Library. See Supplementary Table S4A–D for the colour-coding used in the brain segmentation. Images are from a representative healthy control (F/27 years old).

Volumes of interest (VOIs) were selected using different automatic segmentation tools such as FreeSurfer (v6)^46^ and FIRST-FSL^47^ (Fig. 1). These included cortical gyri, subcortical structures, thalamic nuclei, brainstem substructures and midbrain/cerebellar nuclei.

- Ten cortical gyri were selected from FreeSurfer cortical parcellation: precentral gyrus, caudal middle frontal gyrus, paracentral gyrus and pars triangularis in frontal lobe, postcentral gyrus, inferior parietal gyrus and supramarginal gyrus in parietal lobe, transverse temporal gyrus in temporal lobe and posterior cingulate cortex and isthmus of the cingulate gyrus in the cingulate cortex. Not all the 35 gyri available from FreeSurfer were selected because of overlap artefacts in QSM due to air-tissue boundaries and/or cortical bones.^48^
- Subcortical structures such as caudate, nucleus accumbens, putamen, globus pallidus, thalamus, hippocampus and amygdala were selected using FIRST-FSL.^47^
- Thalamic subnuclei were identified using an atlas-based approach developed by Brun *et al*.^49^ (https://github.com/arnaudletroter/7TAMIBrain). This atlas was generated using high-resolution MP2RAGE images acquired at 7 Tesla from 30 healthy control subjects, resulting in a template in MNI152 space comprising 24 deep GM nuclei, 14 of which correspond to thalamic subregions. Following the authors’ recommended procedure, we registered the atlas template to our MPRAGE images using Advanced Normalization Tools (ANTs; http://stnava.github.io/ANTs). The thalamic nuclei were subsequently categorized into four major groups: anterior, medial, ventral and pulvinar. Supplementary Fig. S1 shows an example of the thalamic nuclei overlaid on MPRAGE and QSM images from a representative participant.
- The brainstem and its substructures (midbrain, pons and medulla) were selected from Freesurfer^50^; in the Supplementary material, brainstem sub-segmentation is shown overlaid to MPRAGE and QSM in a representative participant (Supplementary Fig. S2).
- Substantia nigra (SN), red nuclei (RN) and dentate nuclei (DN) were identified on a study-specific χ-enhanced atlas, constructed in the MNI152 space. QSM images were registered to the corresponding MPRAGE images using ANTs (http://stnava.github.io/ANTs) and exploiting linear registration of MPRAGE to MNI152 space derived using FLIRT (https://fsl.fmrib.ox.ac.uk/fsl/fslwiki/FLIRT). Using mrview from MRtrix3,^51^ we manually drew SN, RN and DN on the atlas; the ROIs were back-registered to the native space for each subject.

### Statistical analysis

Demographics were compared between two groups, using *t*-tests for normally distributed continuous variables; otherwise, Wilcoxon tests were applied. We did not consider E3 class patients because of the limited number and the poor quality of susceptibility images. VOI-based analysis was performed to compare the median χ and volume distributions of HC and DM1 samples and between E1 and E2 patient categories. Values of left and right hemisphere were averaged. Median χ values were corrected by age assuming a linear increase in the control group,^52,53^ and volume was corrected by total intracranial volume using the proportional method.^54^ Correlations between total volume of each structure and χ values were not significant on Spearman’s test, so we did not correct susceptibility values within the VOIs for their volume.

The one-sample Kolmogorov–Smirnov test was used to check the normality of the distributions; as χ and volumes were not normally distributed in any of the structures, the non-parametric Kruskal–Wallis test was used (*P < 0.05 and **P < 0.01). Spearman’s test was used to evaluate potential correlations between χ values and clinical data (*P < 0.05 and **P < 0.01).

For all the tests, age and sex were considered as covariates of no interest and Bonferroni correction was performed within each domain based on the number of VOIs tested.

The statistical analysis was carried out using Python (v. 3.7.6).

## Results

There were no problems encountered in MR acquisition, and VOI segmentation was successful in all cases. Results were analysed for all E1 and E2 patients (*n* = 34) and healthy controls (*n* = 35).

### Cortical gyri

Susceptibility and volume distributions were evaluated in the selected gyri and compared between HC and DM1 groups; results are summarized in Table 3, left side.

**Table 3:** Trend and significances in differences in χ distribution and volume of cortical and subcortical structures in DM1 patients compared to HCs

	Cortical gyri					Subcortical structures			
	Median χ			Volume		Median χ			Volume
PrCG	*	↑	**	↓	Cau	ns	↑	ns	↑
CMFG	**	↑	*ns*	↓	Acc	ns	↓	ns	↓
PCG	*ns*	↑	*	↓	Put	ns	↓	ns	↓
PT	**	↑	*ns*	↓	GP	ns	↓	ns	↑
PoCG	*	↑	*	↓	SN	ns	↓	ns	↑
IPG	**	↑	**	↓	RN	ns	↑	ns	↑
SMG	**	↑	**	↓	Th	*	↑	ns	↑
TTG	**	↑	**	↓	Hipp	ns	↑	ns	↑
PCC	*ns*	↑	*ns*	↑	Amy	ns	↑	ns	↑
ICG	**	↑	*ns*	↑	DN	ns	↑	ns	↑
					Brainstem	**	↑	ns	↑

Susceptibility and volume parameters comparison between DM1 and HCs (↑ = χ increase in DM1 group, ↓ = χ decrease in DM1 group) in cortical gyri (on the left) and in subcortical structures (on the right). ROI-based analysis was performed (Kruskal–Wallis test, *P < 0.05, **P < 0.01).

G, gyrus; PrCG, precentral G; CMFG, caudal middle frontal G; PCG, paracentral G; PT, pars triangularis; PoCG, postcentral G; IPG, inferior parietal G; SMG, supramarginal G; TTG, transverse temporal G; PCC, posterior cingulate cortex; ICG, isthmus of the cingulate gyrus; Cau, caudate; Acc, accumbens; Put, putamen; GP; globus pallidus; SN, substantia nigra; RN, red nucleus; Th, thalamus; Hipp, hippocampus; Amy, amygdala; DN, dentate nucleus; ns, not significant.

Compared with HCs, in the DM1 group, median χ was significantly higher in several cortical gyri, in particular in PrCG (*P* = 0.016), CMF (*P* = 0.001), PT (*P* = 0.005), PoCG (*P* = 0.044), IPG (*P* = 0.005), SMG (*P* = 0.002), TTG (*P* = 0.002) and ICG (*P* = 0.006); some gyri also showed significant atrophy (PrCG, with *P* = 0.005; PCG, with *P* = 0.010; PoCG, with *P* = 0.012; IPG, with *P* = 0.003; SMG, with *P* = 0.002; TTG, with *P* = 0.001).

Further analysis was performed to compare E1 and E2 classes; no significant differences emerged. χ and volume values for HC, DM1, E1 and E2 groups are reported in the Supplementary material (Supplementary Table S1).

### Subcortical structures

**Figure 2:**
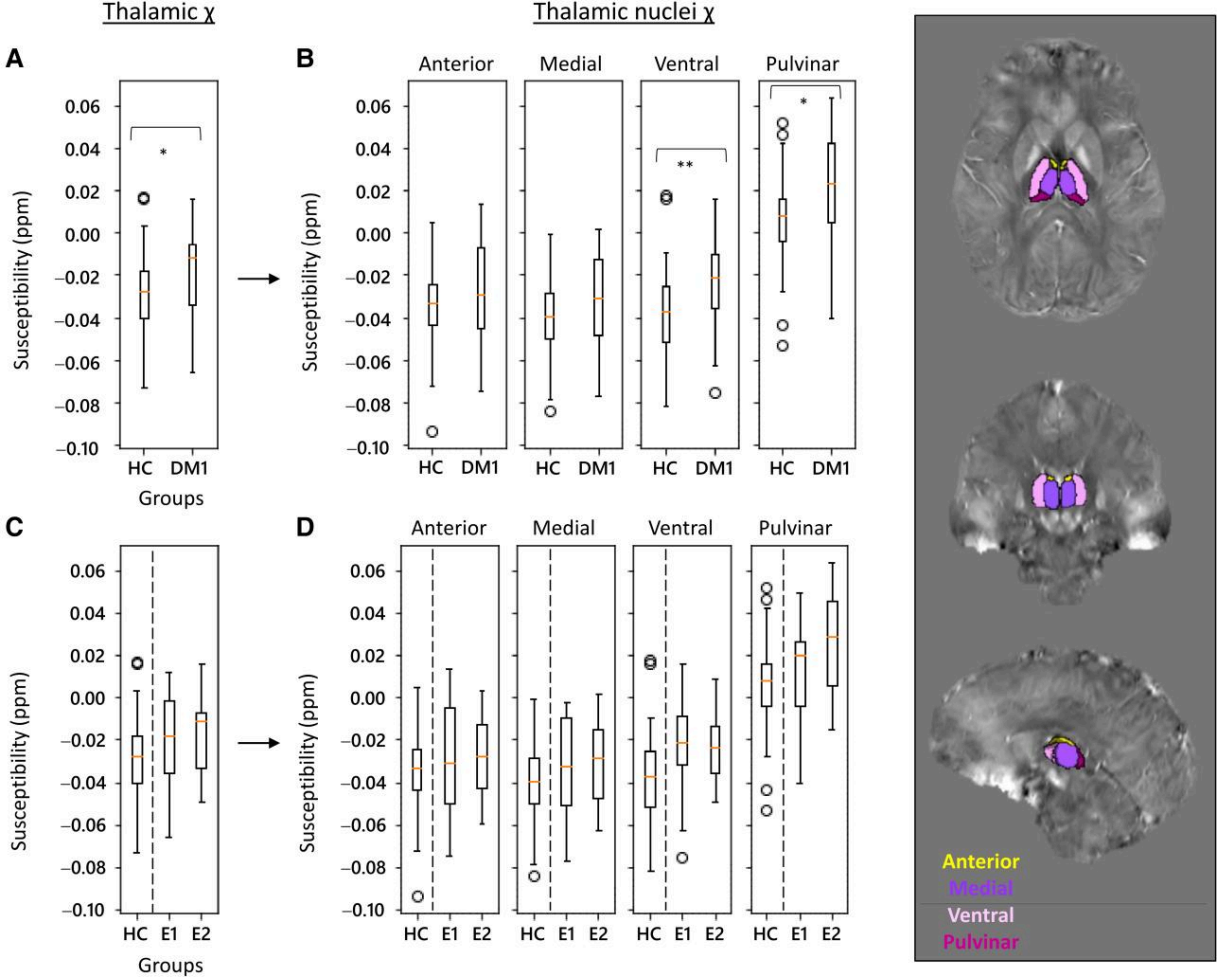
**Thalamic χ distributions in HC and DM1 (E1 and E2 classes).** Distributions were evaluated by comparing the HC cohort with the whole DM1 group (**A**) and distinguishing E1 and E2 classes (**C**). In each panel, the left-hand plot (**A** and **C**) relates to the entire structure, with each thalamic nucleus plotted to the right (**B** and D). VOI-based analysis was performed (**A** and **C**: *N* = 35, 34; **B** and **D**: *N* = 35, 20, 14; Kruskal–Wallis test, *P < 0.05, **P < 0.01). On the right, representative images of thalamic nuclei segmentation (HC, F/26 years old).

**Figure 3:**
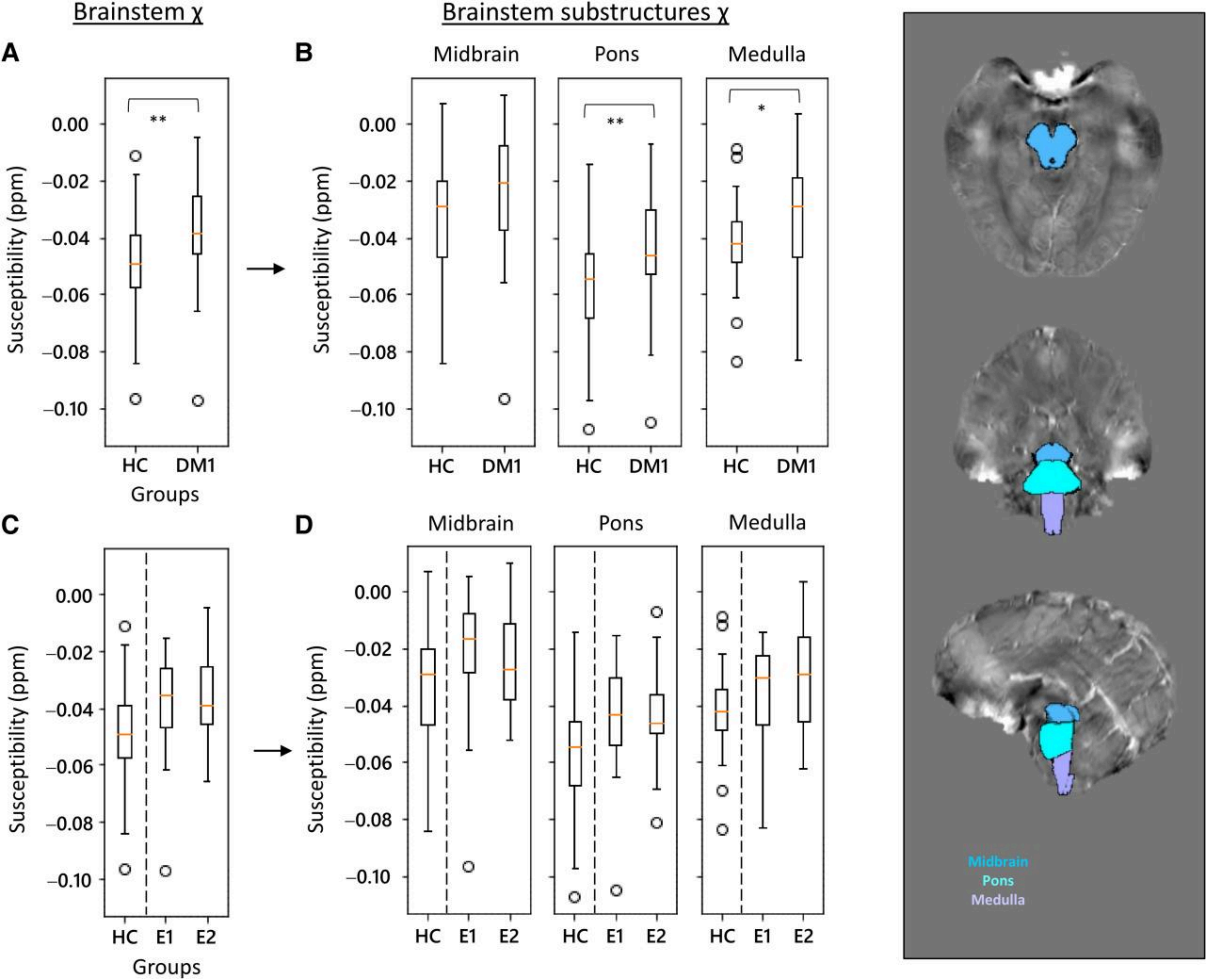
**χ distributions of the brainstem in HC and DM1 (E1 and E2 classes).** Distributions were evaluated comparing the control cohort with the entire DM1 group (**A**) and distinguishing E1 and E2 classes (**C**). In each panel, the left-head plot (**A** and **C**) relates to the entire structure, with plots of substructures (midbrain, pons and medulla) shown on the right (**B** and **D**). ROI-based analysis was performed (**A** and **C**: *N* = 35, 34; **B** and **D**: *N* = 35, 20, 14; Kruskal–Wallis test, *P < 0.05, **P < 0.01). On the right, representative images of brainstem segmentation (HC, F/26 years old).

Susceptibility distribution and volume values were also investigated in subcortical structures, comparing DM1 with HCs. Statistical comparisons with HCs were at first conducted considering the whole DM1 cohort and then subdivided into E1 and E2 genetic classes. χ and volume values for HC, DM1, E1 and E2 groups are reported in the Supplementary material (Supplementary Table S2). First, statistical outcomes are presented in Table 3 (right side). Compared to HCs, the whole DM1 group shows a significant increase of bulk χ in the thalamus (*P* = 0.020) and brainstem (*P* = 0.003) (Figs 2A and 3A), but no significant volume changes. Bar plots with χ and volume distributions in all the subcortical structures for DM1 and HC are shown in the Supplementary material (Supplementary Fig. S3). Although χ and volume distributions were not significantly different among HCs, E1 and E2 groups, E2 displayed a higher bulk susceptibility in caudate, thalamus (Fig. 2D) and amygdala.

### Thalamic nuclei

χ values were analysed in the anterior, medial, ventral and pulvinar nuclei of the thalamus (Fig. 1; Supplementary Fig. S1). Their χ values are reported in the Supplementary material (Supplementary Table S3). Comparisons of HC and DM1 (or E1 and E2) are reported in Fig. 2C and D and Table 4 (upper part). A significant increase in susceptibility was found in the ventral and pulvinar nuclei. No significant differences in χ values were found between the two genetic classes.

**Table 4:** Trend and significances in differences in χ distribution of thalamus and brainstem sub-units in DM1 patients compared to HCs and in DM1-E1 compared to DM1-E2

		HC versus DM1		E1 versus E2	
		*P*-value	χ in DM1	*P*-value	χ in E2
Thalamus	Total	*	↑	ns	↑
	Anterior	ns	↑	ns	↑
	Medial	ns	↑	ns	↑
	Ventral	**	↑	ns	↓
	Pulvinar	*	↑	ns	↑
Brainstem	Total	**	↑	ns	↓
	Midbrain	ns	↑	ns	↓
	Pons	**	↑	ns	↓
	Medulla	*	↑	ns	↑

On the left side, comparison of DM1 and HC (↑ = χ increase in DM1 group, ↓ = χ decrease in DM1 group); on the right side, comparison of E1 and E2 (↑ = χ increase in E2 class, ↓ = χ decrease in E2 class). ROI-based analysis was performed (Kruskal–Wallis test, *P < 0.05, **P < 0.01).

ns, not significant.

### Brainstem sub-units

We analysed the midbrain, pons and medulla separately and as parts of the brainstem (Fig. 1; Supplementary Fig. S2). χ values for brainstem sub-segmentation are reported in the Supplementary material (Supplementary Table S3).

Statistical comparisons are reported in the lower part of Table 4 and in Fig. 3. Compared to HCs, pons, medulla and the entire brainstem showed a significant χ increase in DM1 that was higher in the pons (Fig. 3A and B). No χ values differences emerged when comparing brainstem units between E1 and E2 patients (Fig. 3C and D).

### Clinical correlations

Correlation analyses were conducted to reveal possible associations between χ values in subcortical structures and clinical data. Table 5 reports correlation results in the DM1 group. In the table, the direction of severity (i.e. ↑ = increase or ↓ = decrease) for each clinical parameter is reported (please refer to the pathological cut-offs indicated in Table 2); an increase of susceptibility is related to an increase of iron accumulation, indicating a possible biomarker of disease severity. Main findings were the following:

- Higher χ values correlated with a younger onset age in the thalamus—specifically, in the medial and pulvinar nuclei considering the sub-segmentation—and in the brainstem.
- χ values within thalamus and amygdala are linked with disability scores. Thalamic χ in the pulvinar positively correlated with three out of four domains of NIFDS (motor, myotonic and daily life activities); the correlation in the motor domain remained true in the E2 class individually. Amygdala χ correlated with motor and daily life domain of NIFDS, as well as in the total counts; as before, this was true also in the individual E2 class, where, additionally, a correlation between amygdala χ and the neuropsychological domain scores was observed.
- Pneumological data did not correlate with any structures considering the patient sample as a whole; Although Table 5 reports only differences between DM1 group and HCs, there was a positive correlation in the E2 class with the PaCO_2_ within the anterior and medial thalamic nuclei and the medulla oblongata.
- Within the cardiological data, correlations were found between the PR interval length and brain structures, namely, whole thalamus; medial, ventral and pulvinar nuclei; medulla oblongata; amygdala and putamen. Thalamic, medial and pulvinar χ positively correlated within E1 and E2 classes individually; amygdala χ also correlated with the E2 class.
- There were correlations with the index of central apnoea in the thalamic medial nucleus, the brainstem, the hippocampus and the putamen.

**Table 5:** Trend and significances in correlations between χ values and clinical data in DM1 patient cohort

		Thalamus	Brainstem	Others
Onset age (↓)		(−) Th (*) Th-M (*) Th-P (*)	(−) Br (*)	*ns*
Neurological	MIRS (↑)	ns	ns	ns
evaluation	NIFDS (NP) (↑)	ns	ns	ns
	NIFDS (Mo) (↑)	(+) Th-P (*)	ns	(+) Amy (*)
	NIFDS (My) (↑)	(+) Th-P (**)	ns	ns
	NIFDS (DL) (↑)	(+) Th-P (*)	ns	(+) Amy (*)
	NIFDS (tot) (↑)	ns	ns	(+) Amy (*)
	ESS (↑)	ns	ns	ns
Pneumological	FVC (↓)	ns	ns	ns
evaluation	PaCO_2_ (↑)	ns	ns	ns
	PaO_2_ **(**↓)	ns	ns	ns
Cardiological	PR int length (↑)	(+) Th (**) Th-M (**) Th-V (**)	(+) Br-Mo (*)	(+) Amy (*) Put (**)
evaluation		Th-P (**)		
	QRS int length (↑)	ns	ns	ns
	EF (↓)	*ns*	ns	ns
Sleep recordings	ODI (↑)	*ns*	ns	ns
	OAHI (↑)	*ns*	ns	ns
	CAHI (↑)	(+) Th-M (*)	(+) Br (*)	(+) Hipp (*) Put (*)

Spearman’s test (*P < 0.05, **P < 0.01) was used to evaluate possible correlations between clinical data from neurological, pneumological, cardiological and sleep evaluations and χ values in subcortical structures. For each clinical parameter, the arrow in brackets (↑↓) indicates an increase or decrease of the clinical scores (please refer to the pathological cut-offs indicated in Table 2). For each subcortical unit, the sign in brackets (+/−) indicates the direction of the χ values relationship with clinical data.

Th, thalamus; Th-A, anterior; Th-M, medial; Th-V, ventral; Th-P, pulvinar; Br, brainstem; Br-Mi, midbrain; Br-P, pons; Br-Mo, medulla oblongata; Cau, caudate; Acc, accumbens; Put, putamen; GP, globus pallidus; SN, substantia nigra; RN, red nucleus; Hipp, hippocampus; Amy, amygdala; DN, dentate nucleus; MIRS, Muscular Impairment Rating Scale; NIFDS, Neuromuscular Impairment Function and Disability Scale; NP, neuropsychological; Mo, motor; My, myotonia; DL, daily life activity; ESS, Epworth Sleepiness Scale; FVC, forced vital capacity; PaCO_2_, arterial carbon dioxide partial pressure; PaO_2_, arterial oxygen partial pressure; EF, ejection fraction; ODI, oxygen desaturation index; OAHI, obstructive apnoea hypopnoea index; CAHI, central apnoeas hypopnoea index; ns, not significant.

## Discussion

In this study, we used quantitative mapping of magnetic susceptibility as an indirect measure of iron content in brain structures of DM1 (E1 and E2 genetic classes) patients compared to healthy controls. Our aim was 2-fold: to better understand the pathological development of the disease and to find specific imaging biomarkers, since neurodegeneration often presents quantitative and qualitative radiological signs that are sensitive but not specific.

There are several studies reported using advanced MRI techniques to explore SNC involvement in patients with DM.^13,32^ However, to date, only one study has examined susceptibility-based images in DM1,^33^ showing that DM1 patients are characterized by a widespread magnetic susceptibility increase in subcortical structures that is also associated with clinical symptoms such as muscular weakness, daytime sleepiness and specific cognitive deficits. Our results confirm these outcomes, exploring a homogeneous sample of DM1 patients, expanding the susceptibility analysis to numerous cortical and subcortical GM structures and combining imaging data with a comprehensive neurological evaluation.

Our QSM analysis utilizes voxel-wise maps of mean tissue magnetic susceptibility. While these maps do not provide a direct quantification of iron content,^29,30^ susceptibility changes are interpreted primarily as reflecting variations in iron deposition, with consideration given to the specific brain region examined.^32^ Indeed, multiple studies have demonstrated that in deep GM regions, QSM serves as a reliable proxy for iron content due to minimal confounding effects from myelin or other metals, findings supported by post-mortem validation.^31,55-57^ In contrast, within WM, susceptibility alterations reflect a combination of iron and myelin changes, such that increases in susceptibility may indicate iron accumulation, demyelination or a combination thereof.^32^ Previous studies have highlighted cortical differences between DM1 and HC, in particular a widespread cortical atrophy in frontal, temporal, parietal and occipital lobes.^13^ In our study, the reported structural alterations in DM1 brains were associated with significant increase of susceptibility in cortical gyri (Table 3), in particular in the frontal lobe (precentral and caudal middle frontal gyri, pars triangularis), in the parietal lobe (post-central, inferior-parietal and supramarginal gyri), in the temporal lobe (transverse temporal gyrus) and in the mid-posterior cingulate cortex (isthmus of the cingulate cortex).

Increase of susceptibility in cortical gyri is linked to iron accumulation that is in turn related to a worse clinical condition in many neurodegenerative disorders such as amyotrophic lateral sclerosis.^32,58^ A similar hypothesis can be formed for the DM1 disorder. Iron accumulation has been found in cortical areas that are implicated in those sensorimotor and cognitive functions generally compromised in DM1 patients: motor and language skills (frontal lobe), sensory perception (parietal lobe), auditory function (temporal lobe) and memory and awareness (posterior cingulate cortex).^59,60^

Cortical iron dysregulation is a common phenomenon observed in both aging and various neurodegenerative disorders, rendering it unsuitable as a specific biomarker for DM1.^25-28,30-33^ Consequently, we focused our investigation on magnetic susceptibility changes within subcortical structures. Alterations in these regions—particularly the thalamus—appear to display disease-specific patterns. For example, patients with secondary progressive multiple sclerosis (SPMS) exhibit reduced thalamic susceptibility compared to healthy controls.^61^ In contrast, our DM1 cohort demonstrated a significant increase in susceptibility, compared to healthy controls, in both the thalamus, consistent with findings by Ates *et al*.^33^ who attributed iron alterations in *N* = 12 DM1 patients to underlying pathophysiological mechanisms, and the brainstem (Table 3). Furthermore, post-mortem studies combining 7T MR imaging and histopathological analyses have confirmed a positive correlation between susceptibility values and iron concentration in cortical GM and subcortical structures.^31-65^ In our study, the brainstem was one target structure identified comparing susceptibility distributions between DM1 patients and HCs. It plays a pivotal role in conveying communication between the spinal cord and brain and also controls many autonomic functions, such as heart rate, blood pressure and respiratory control during both sleep and wakefulness. Breathing disorders in DM1—including sleep-related breathing disorders—have a complex aetiology, combining both peripheral involvement (respiratory muscle weakness and myotonia) and central respiratory drive dysfunction^66-68^ (see also Poussel *et al*.^69^ and Callus *et al*.^70^ for a debate on the central and/or peripheral involvement).

In our study, χ values were assessed within the three main brainstem structures, midbrain, pons and medulla, and significant differences between DM1 and controls were mainly observed in both pons and medulla, which disclosed higher susceptibility values in DM1 patients along with other nuclei relaying signals from the forebrain to the cerebellum. The pons contains two respiratory centres, the apneustic centre and the pneumotaxic centre, working together to regulate breathing rate and depth; the medulla houses respiratory centres, and its nucleus ambiguus and the dorsal motor nucleus of the vagus nerve are involved in motor functions of swallowing, speech and parasympathetic innervation of the heart and viscera.

The reported brainstem functional properties form a coherent framework for the findings of positive correlations between brainstem iron accumulation and a worsening of cardiological and nocturnal respiratory parameters. Specifically, the medulla’s susceptibility was positively correlated with the PR interval length, an electrocardiographic parameter which indicates a slowing of conduction between the atria and ventricles. It is worth mentioning that the precise mechanisms by which DM1 promotes cardiac conduction system dysfunction are still under investigation. Cardiac fibrosis and fatty infiltration can affect the His–Purkinje system and the sino-atrial and atrioventricular (AV) nodes, providing a substrate for conduction block, ectopic activity and re-entrant arrhythmias.^71,72^ Abnormal splicing of the SCN5A gene demonstrated in DM1 patients has also been implicated in cardiac conduction system disease.^73^ Cardiovascular autonomic nervous system dysfunction (cANS) and its relationship with heart involvement in DM1 patients remain unclear.^74,75^ Clinical and neurophysiological studies, using cardiovascular reflex tests and power spectral analysis (PSA) of heart rate variability (HRV), investigated cardiovascular autonomic function on small groups of DM1 patients, with conflicting results.^76^ Our finding of a positive association between an increased conduction interval and χ increase in central structures should thus be interpreted cautiously; however, together with the results on breath disorders described below, it suggests a possible role for susceptibility change in CNS as a biomarker of DM1 clinical manifestations.

The reported frequency of central apnoeas appeared to increase along with the increased magnetic susceptibility as assessed in the whole brainstem structure. As previously pointed out, sleep disorders are common in DM1 disease and include sleep disordered breathing and fatigue^77^; in accordance with Hamilton *et al*.^78^ who showed an association between altered brain volumetry and alteration of sleep architecture and efficiency in DMs, our findings on an association between magnetic susceptibility in central structures and sleep and respiratory dysfunctions support the hypothesis of a prevalent role of the central structures in the pathogenesis of sleep respiratory disturbances in DM1. Some anatomopathological studies seem to further support a possible involvement of the autonomic nervous system (ANS) in DM, specifically Ono *et al*.^79,80^ observed a loss of catecholaminergic neurons in the medullary reticular formation in DM patients with alveolar hypoventilation, supporting a possible CNS involvement in respiratory dysfunction. Moreover, the association between magnetic susceptibility in the brainstem and sleep anomalies has been recently confirmed by a study from Nepozitek *et al*.^81^ in a cohort of patients with REM sleep behaviour disorder.

The thalamus is a paired structure, relaying peripheral sensory and motor information to distinct areas of the cerebral cortex. It is furthermore involved in the regulation of consciousness, sleep and alertness. Considering the anatomical position and connections within the CNS, its alterations might be associated with impairments, ranging from motor to consciousness deficits. Investigating iron accumulation in specific thalamic nuclei may offer straightforward information about its possible association with clinical outcomes. Note that the thalamus is primarily a GM structure, but it also contains WM for neural connections; however, we can say that the contribution to susceptibility comes from paramagnetic substances and that QSM values are proportional to iron concentrations.^31^

In our study, iron change was analysed in the anterior, medial, ventral and pulvinar thalamic nuclei (Fig. 2B and Table 4). Susceptibility alterations were significant in both ventral and pulvinar nuclei. Ventral thalamic nuclei project to the somatosensory and motor cortices and relay information about movement, tremor and sensory system, while pulvinar nuclei project to the visual cortex and are related to visual processing. Notably, in our DM1 cohort, susceptibility alterations were also found in motor, sensory and visual cortices, all recipients of the thalamic inputs.

Comparing the thalamic susceptibility of DM1 E1 and E2 classes, we found a trend of χ increase that mirrored the nucleotide triplet’s expansion size, in that the E2 class showed higher susceptibility values. This trend was present either when analysing susceptibility in the whole thalamus or in the anterior, medial and pulvinar nuclei individually. Although non-significant, this outcome suggests the existence of a possible association between the increase of thalamic susceptibility and the worsening of clinical conditions (Fig. 2C and D).

This hypothesis was strengthened by the finding of an association between thalamic susceptibility and clinical data (Table 5). First, we found that susceptibility values were negatively correlated with onset age, suggesting that DM1 patients with an earlier onset present higher iron concentration levels, both in the thalamus as a whole and in the medial and pulvinar nuclei separately. Additionally, thalamic pulvinar susceptibility correlated with clinical tests quantifying DM1 disability, specifically: (i) positive correlations between iron levels and NIFDS motor, myotonia and daily life activities subscales showed that an increase in thalamic χ values was related to a worsening of neuromuscular impairments and of their impact on basic life activities. However, as notably the genesis of DM1 muscular weakness and myotonia has peripheral origins, motor deficits can only be indirectly related to thalamic pulvinar χ. Indeed, as significant correlations with NIFDS disability scores were only found among E2 patients, a genetic class characterized by an earlier disease onset associated with higher triplet expansion size compared to E1, the reported association can be interpreted as mainly driven by the effect of a worsening motor impairment over longer disease duration time; (ii) in all DM1 cohort and E1/E2 genetic subgroups, thalamic susceptibility was positively correlated with indices of cardiac conduction abnormalities and arrhythmia (PR interval length); (iii) χ values of medial thalamic nuclei were positively correlated with the occurrence of breathing disorders such as central sleep apnoea number (CAHI), associating higher values of susceptibility to a higher frequency of central sleep apnoeas—no other correlations were found between subcortical structures and two other indices of apnoeas (ODI and OAHI), reporting central and non-central episodes accordingly.

Thalamus is considered a crucial hub for sensory signals processing and it acts as a relay station that most sensory information must pass before reaching the cortex.^82^ Medial thalamic nuclei have been recently proposed as a component of the respiratory neural network responding to respiratory load stimuli, thus relaying respiratory mechanical information to the cerebral cortex for the conscious awareness of breathing.^83^ While eupneic breathing is usually not consciously perceived, modulation of breathing (hyperpnea or inspiratory occlusions) activates distinct cortical and subcortical structures among which medial thalamus.^84,85^ The finding of a correlation between iron accumulation in medial thalamic nuclei and central sleep apnoea suggests that breathing dysfunctions during the sleep might be related to CNS alterations.

Other deep GM structures seem to be involved in the central pathogenesis of DM1: (i) susceptibility in the amygdala exhibited correlations with motor and daily life items in the NIFDS within the DM1 group and the E2 class individually, and additionally, it correlated with the PR interval length within the entire DM1 group and the E2 class; (ii) putaminal susceptibility was linked with the PR interval length and the number of central apnoeas; (iii) susceptibility within the hippocampus correlated with the number of central apnoeas—hippocampus activity is coordinated by respiration^86^ but also indirectly implicated in the modulation of augmented breath.^87^

Together, the presented results showed that central apnoea frequency correlates with iron accumulation in the brainstem and that conduction changes may be associated with increased magnetic susceptibility in central structures, thus suggesting a potential involvement of central mechanisms in cardiac and respiratory alterations in DM1 patients. While these results provide valuable insights into DM1 pathophysiology, their interpretation should be cautious with regard to the systemic nature of the DM disease.

This study has several limitations. First, the sample size was still relatively small and did not include DM1 patients belonging to E3 genetic class: an incomplete cross-sectional study design may not have captured the whole picture on the association between iron accumulation in SNC and the severity of clinical impairment. Second, while our study focused on within-group associations in the DM1 cohort, future studies should include clinically assessed controls in order to enhance the specificity and interpretability of the QSM findings. Moreover, a cognitive assessment using a formal neuropsychological battery would be recommended to better apprehend the complexity of cognitive impairment in DM1. Third, bulk susceptibility is a composite measure; future analyses would benefit of χ-separation methods to disentangle paramagnetic and diamagnetic sources contributions to susceptibility changes or approaches with heightened sensitivity to paramagnetic signal changes.^88-92^ Fourth, a longitudinal study with a larger sample size would be necessary to further explore the temporal relationship of DM on brain QSM values and clinical signs. Finally, the validity of the data depends on the appropriateness of each part of the analysis chain, in particular the accuracy of ROIs defined on atlas defined in the MNI 152 space not participants’ own native space.

## Conclusion

The main finding of our study is that beyond well-known cortical atrophy, DM1 patients present higher levels of magnetic susceptibility changes indicative of iron content in both cortical and subcortical structures. The latter areas, specifically thalamus and brainstem, showed the most pronounced changes and are also involved in some of the autonomic functions (breathing control and cardiac conduction regulation) that show a progressive impairment over time in DM1 patients. Moreover, the findings that iron accumulation is correlated with both disease duration over time and a worsening of the clinical conditions shed new light on the central origins of the autonomic nervous system dysfunctions in DM1.

Together, the presented results showed that changes in CNS function are associated with iron accumulation in subcortical structures as detected by QSM. Magnetic susceptibility changes in subcortical structures constitute candidate biomarkers for disease detection and progression in DM1.

## Supplementary Material

fcag017_Supplementary_Data

## Data Availability

The data that support the findings of this study are available from the corresponding author, upon reasonable request. Regarding the source code for any specialized, in-house scripts or programs, that are necessary for the reproduction of results, scripts for image registration and radiomic feature extraction are hosted in the Zenodo repository ‘MRI dataset for susceptibility-based radiomic feature extraction in healthy controls and patients with multiple sclerosis’ (https://zenodo.org/records/10931121).
