# Supplementary material for: Quantitative susceptibility mapping in myotonic dystrophy: clinical relevance of subcortical iron accumulation
Source: Brain Commun. 2026 Jan 20;8(1):fcag017. doi: 10.1093/braincomms/fcag017 (PMC12875119; doi:10.1093/braincomms/fcag017)
Supplement: fcag017_Supplementary_Data [file fcag017_supplementary_data.docx]

**Supplementary Materials**

**Supplementary Figure S1:**


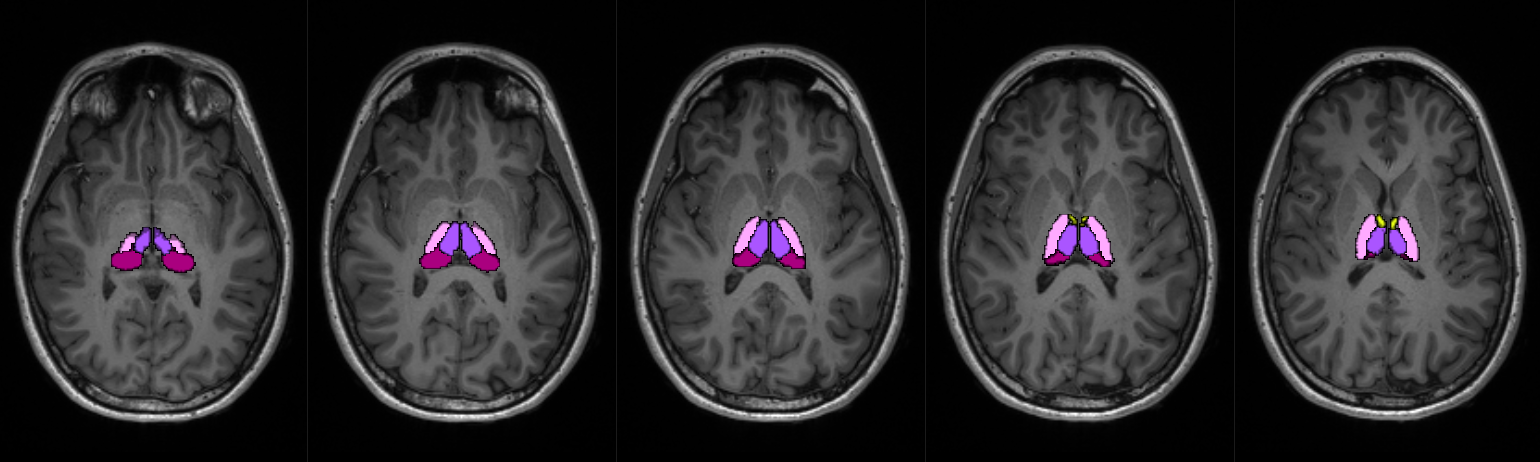

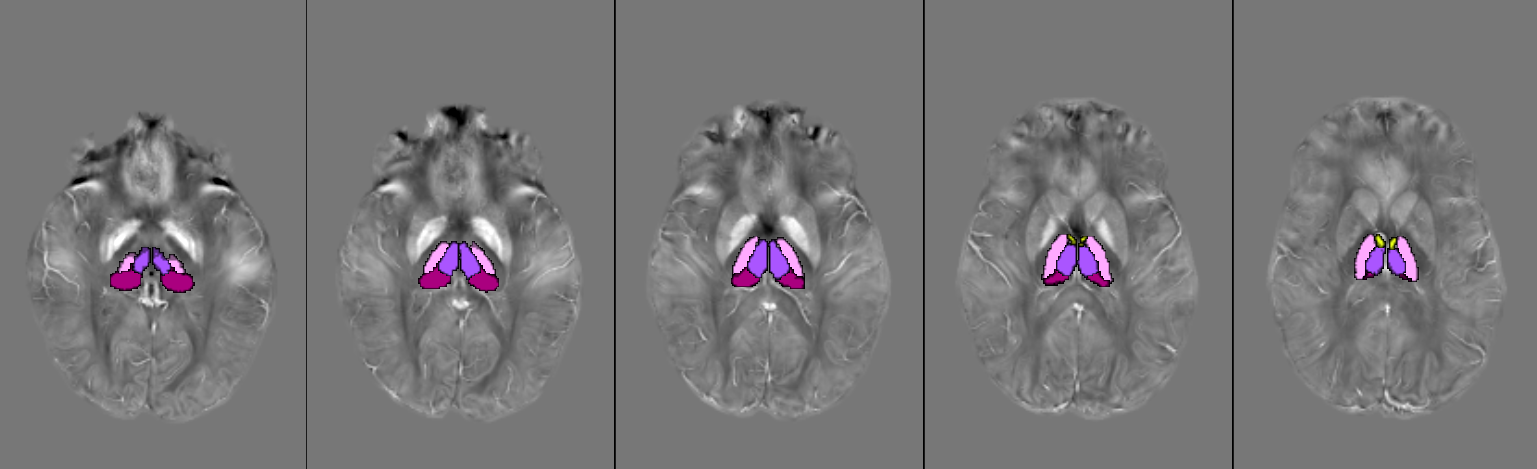

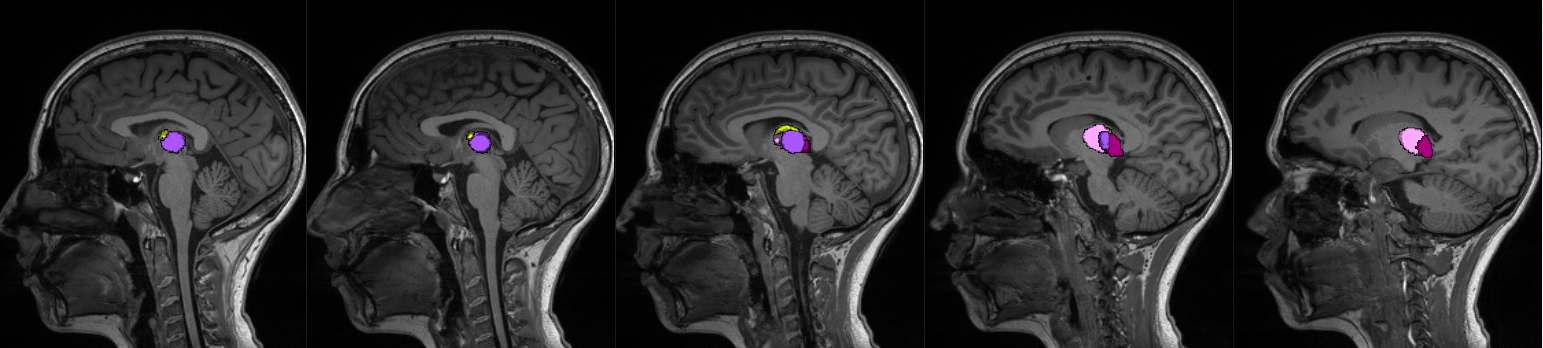

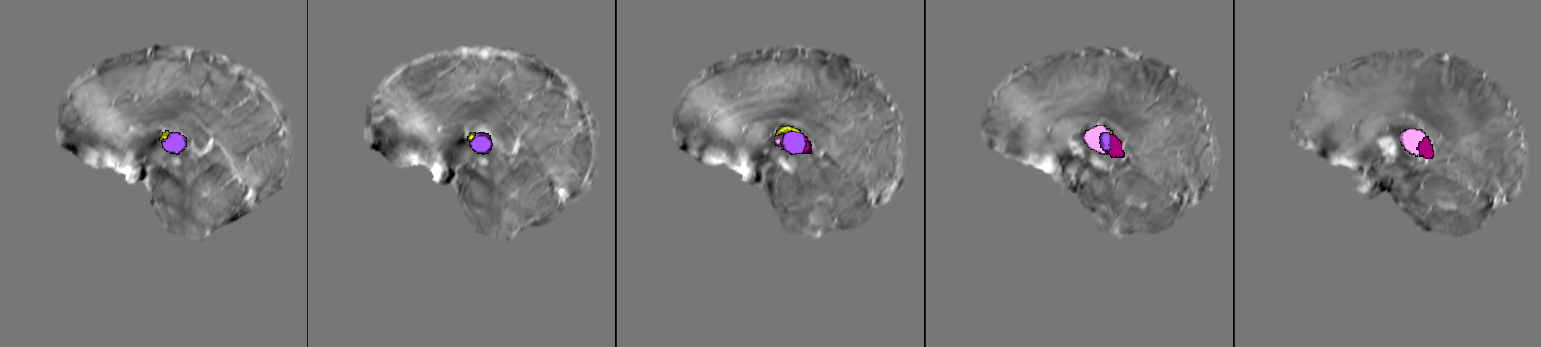

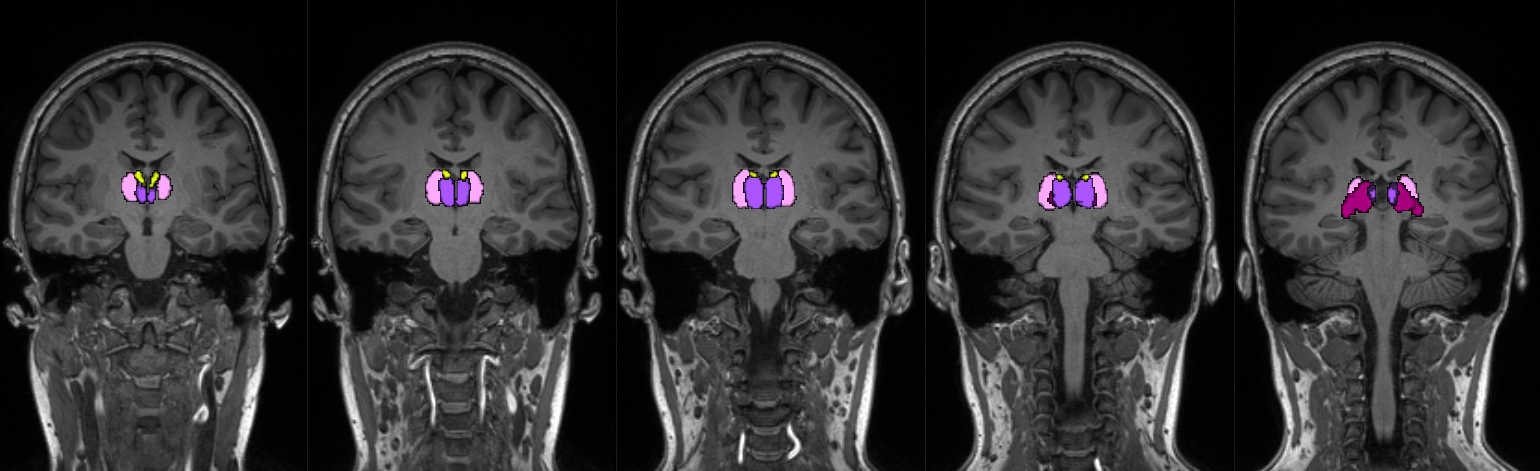

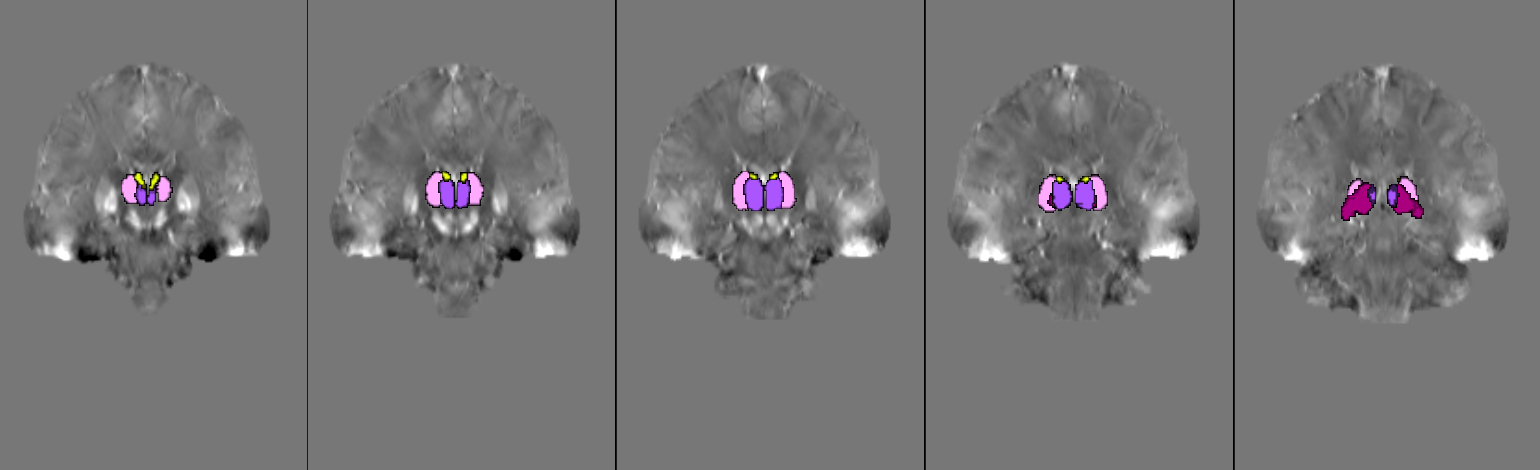


Thalamic segmentation (Anterior, Medial, Ventral and Pulvinar nuclei)

QSM

A

B

MPRAGE

Thalamic segmentation in anterior, medial, ventral and pulvinar nuclei overlaid on MPRAGE (A) and QSM (B) images of a representative subject of the study cohort (F/27yo). The colour-coding for thalamic nuclei segmentation is provided in Supplementary Tab. S4 (B).Thalamic nuclei segmentation was performed using the template from Brun, et al. (2022)

**Supplementary Figure S2:**


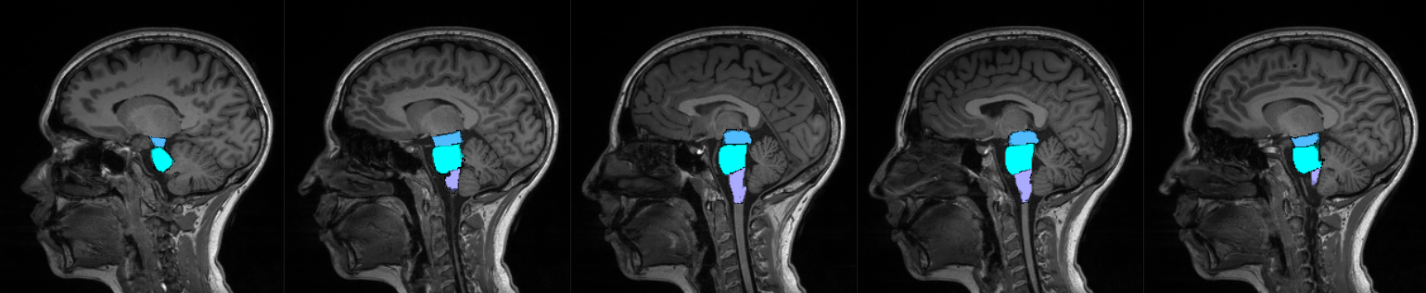

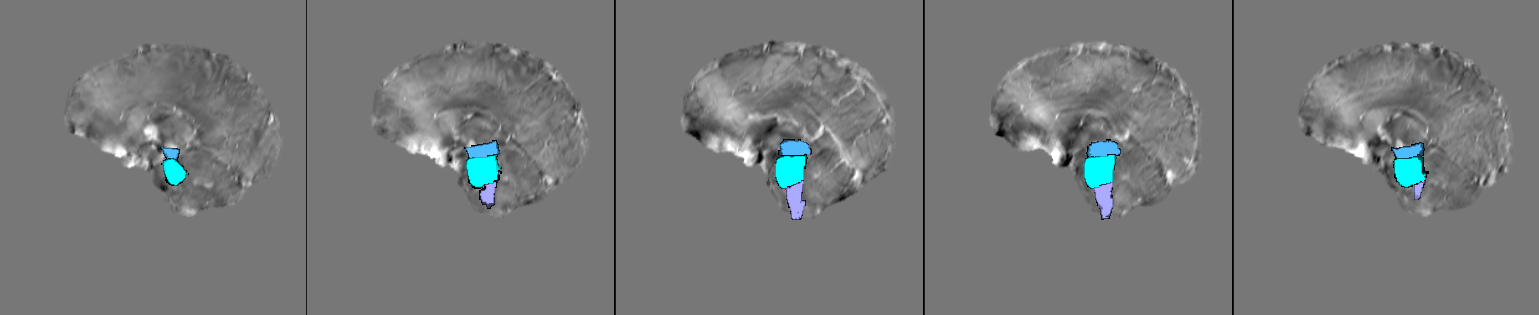

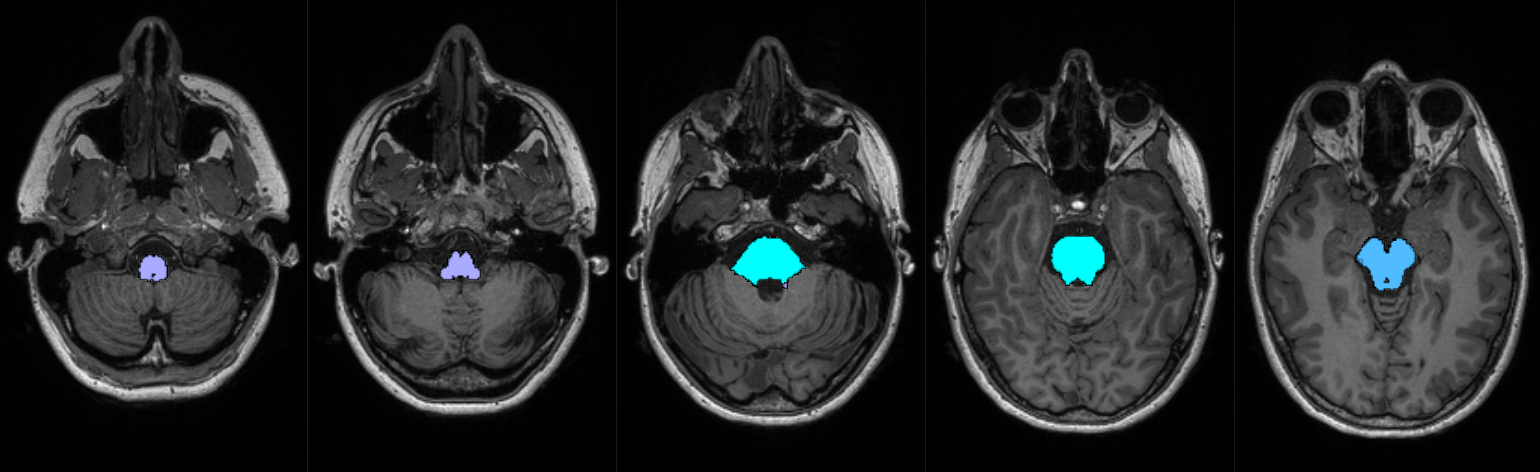

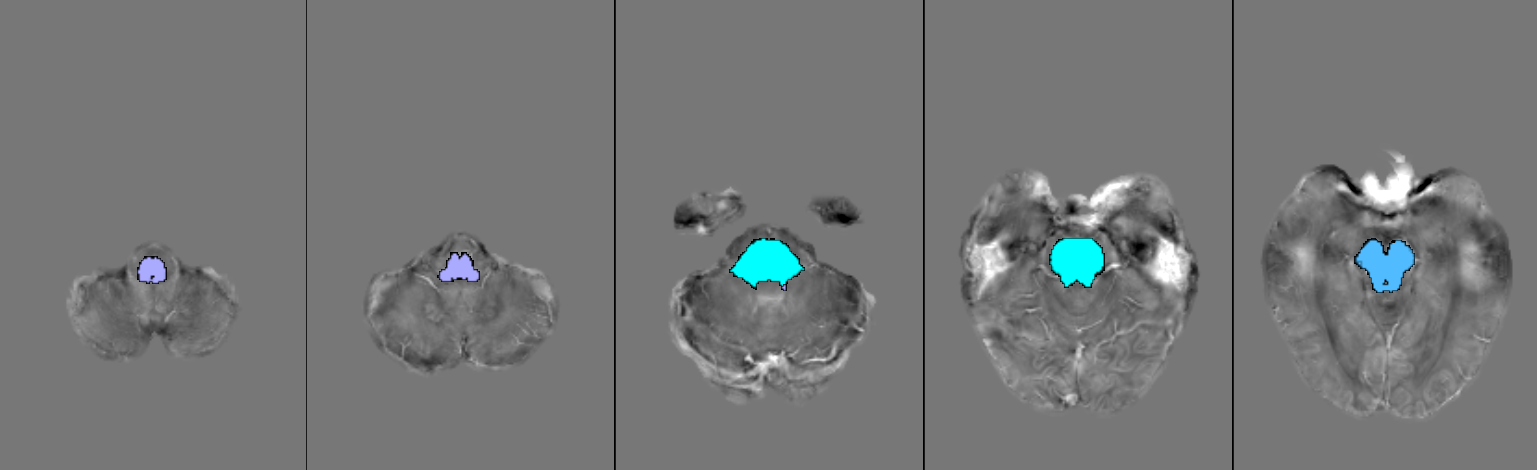

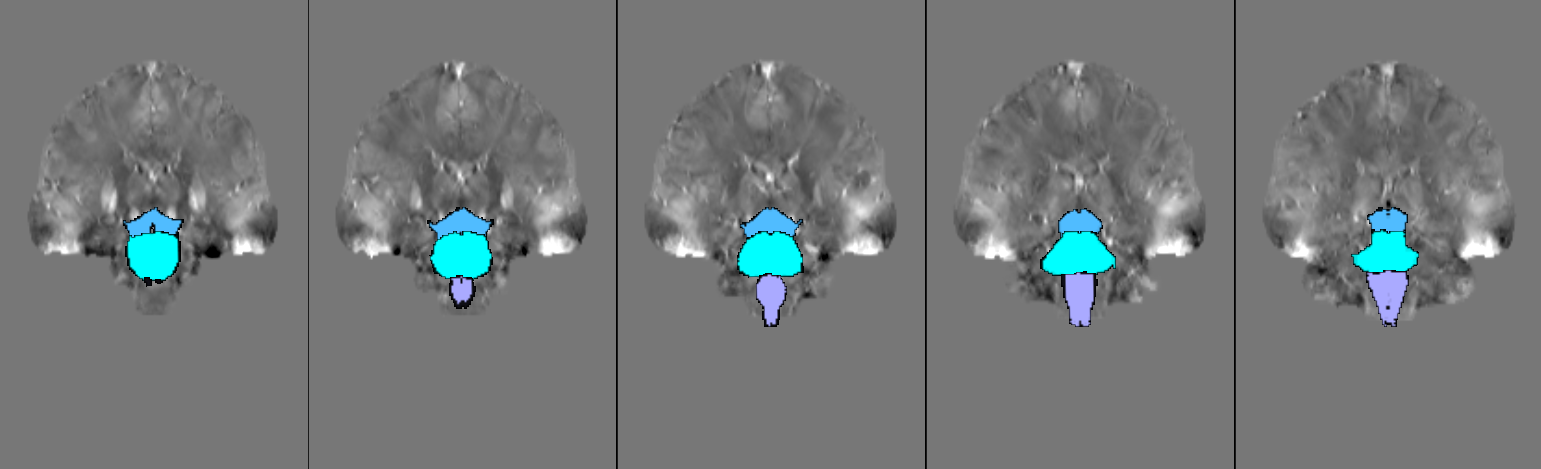

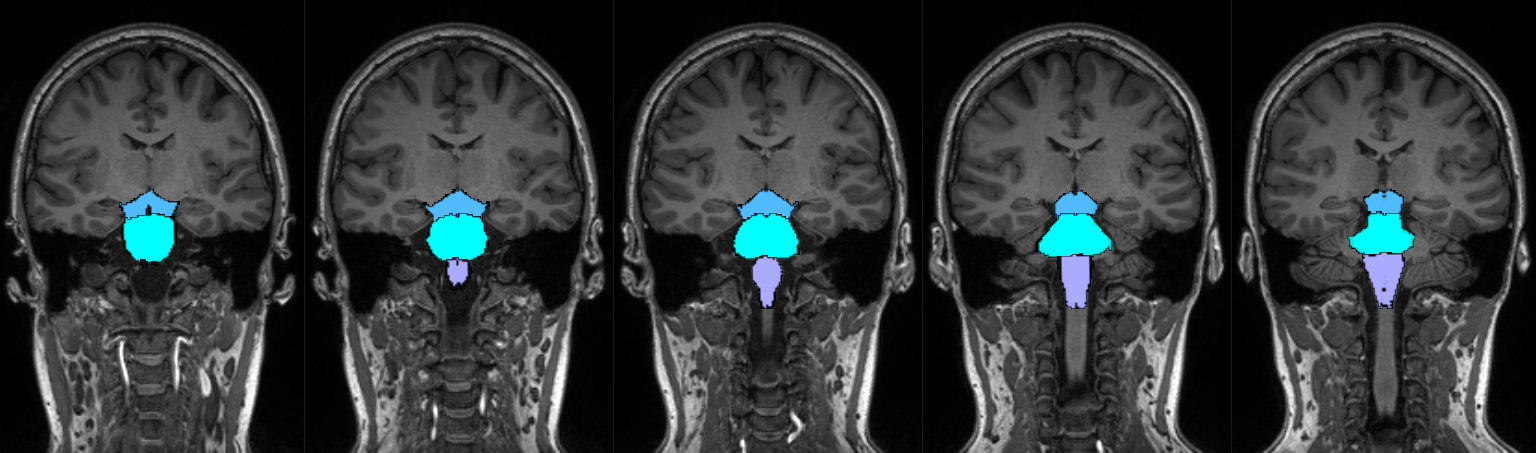


Brainstem segmentation (Midbrain, Pons, Medulla)

MPRAGE

QSM

A

B

Brainstem segmentation in midbrain, pons and medulla from FreeSurfer, overlaid on MPRAGE (A)and QSM (B) images of a representative subject of the study cohort (F/27yo). See Supplementary Tab. S4 (C) for the colour-coding used in the brainstem segmentation.

**Supplementary Table S1:**

|  | | **Median χ (ppm)** | | | |
| --- | --- | --- | --- | --- | --- |
|  | | **HC** | **DM1** | **E1** | **E2** |
| **Frontal L** | **PrCG** | -0.020 ± 0.008 | -0.015 ± 0.008 | -0.016 ± 0.008 | -0.014 ± 0.008 |
|  | **CMFG** | -0.020 ± 0.006 | -0.013 ± 0.006 | -0.016 ± 0.005 | -0.011 ± 0.006 |
|  | **PCG** | -0.022 ± 0.009 | -0.018 ± 0.011 | -0.014 ± 0.011 | -0.020 ± 0.010 |
|  | **PT** | -0.013 ± 0.008 | 0.000 ± 0.009 | 0.001 ± 0.009 | 0.000 ± 0.009 |
| **Parietal L** | **PoCG** | -0.016 ± 0.010 | -0.010 ± 0.011 | -0.007 ± 0.009 | -0.010 ± 0.010 |
|  | **IPG** | -0.010 ± 0.009 | -0.002 ± 0.008 | -0.000 ± 0.010 | -0.004 ± 0.007 |
|  | **SMG** | -0.010 ± 0.008 | 0.002 ± 0.009 | 0.005 ± 0.008 | 0.001 ± 0.007 |
| **Temporal L** | **TTG** | 0.007 ± 0.013 | 0.021 ± 0.009 | 0.019 ± 0.008 | 0.024 ± 0.010 |
| **Cingulate C** | **PCC** | -0.035 ± 0.008 | -0.029 ± 0.007 | -0.026 ± 0.006 | -0.031 ± 0.008 |
|  | **ICG** | -0.004 ± 0.012 | 0.005 ± 0.011 | 0.008 ± 0.009 | 0.002 ± 0.008 |
|  |  |  |  |  |  |
|  |  | **Volume (a.u.)** | | | |
|  |  | **HC** | **DM1** | **E1** | **E2** |
| **Frontal L** | **PrCG** | 1.134 ± 0.049 | 1.033 ± 0.054 | 1.022 ± 0.023 | 1.062 ± 0.078 |
|  | **CMFG** | 0.491 ± 0.046 | 0.450 ± 0.036 | 0.445 ± 0.034 | 0.452 ± 0.034 |
|  | **PCG** | 0.321 ± 0.014 | 0.298 ± 0.021 | 0.309 ± 0.017 | 0.289 ± 0.016 |
|  | **PT** | 0.330 ± 0.034 | 0.314 ± 0.032 | 0.309 ± 0.028 | 0.321 ± 0.038 |
| **Parietal L** | **PoCG** | 0.802 ± 0.051 | 0.749 ± 0.053 | 0.755 ± 0.046 | 0.749 ± 0.072 |
|  | **IPG** | 1.164 ± 0.088 | 1.066 ± 0.084 | 1.019 ± 0.046 | 1.095 ± 0.090 |
|  | **SMG** | 0.947 ± 0.054 | 0.860 ± 0.056 | 0.845 ± 0.056 | 0.874 ± 0.044 |
| **Temporal L** | **TTG** | 0.093 ± 0.009 | 0.081 ± 0.010 | 0.090 ± 0.009 | 0.080 ± 0.006 |
| **Cingulate C** | **PCC** | 0.251 ± 0.021 | 0.259 ± 0.015 | 0.262 ± 0.021 | 0.256 ± 0.014 |
|  | **ICG** | 0.219 ± 0.017 | 0.219 ± 0.015 | 0.218 ± 0.012 | 0.219 ± 0.019 |

Median χ (top) and volume (bottom) values (median ± mean absolute deviation) in HC, DM1, E1 and E2 groups in the analyzed cortical gyri. Median χ is expressed in parts per million (ppm). Volumes were corrected by total intracranial volume using the proportional method, so they are in arbitrary units (a.u.). (L = lobe; C= cortex; G = Gyrus; PrCG = PreCentral G; CMFG = Caudal Middle Frontal G; PCG = ParaCentral G; PT = Pars Triangularis; PoCG = PostCentral G; IPG = Inferior Parietal G; SMG = SupraMarginal G; TTG = Transverse Temporal G; PCC = Posterior Cingulate Cortex; ICG = Isthmus of the Cingulate Gyrus)

**Supplementary Table S2:**

|  | **Median χ (ppm)** | | | | |
| --- | --- | --- | --- | --- | --- |
|  | **HC** | **DM1** | | **E1** | **E2** |
| **Cau** | 0.026 ± 0.011 | 0.026 ± 0.010 | 0.026 ± 0.012 | | 0.026 ± 0.010 |
| **Acc** | 0.004 ± 0.017 | 0.004 ± 0.021 | -0.003 ± 0.022 | | 0.006 ± 0.021 |
| **Put** | 0.023 ± 0.016 | 0.020 ± 0.016 | 0.025 ± 0.015 | | 0.017 ± 0.015 |
| **GP** | 0.100 ± 0.021 | 0.080 ± 0.023 | 0.085 ± 0.026 | | 0.078 ± 0.020 |
| **SN** | 0.060 ± 0.020 | 0.060 ± 0.022 | 0.072 ± 0.018 | | 0.056 ± 0.020 |
| **RN** | 0.041 ± 0.026 | 0.058 ± 0.018 | 0.069 ± 0.028 | | 0.050 ± 0.013 |
| **Th** | -0.027 ± 0.013 | -0.011 ± 0.017 | -0.018 ± 0.020 | | -0.011 ± 0.019 |
| **Hipp** | -0.032 ± 0.008 | -0.024 ± 0.013 | -0.023 ± 0.012 | | -0.029 ± 0.014 |
| **Amy** | -0.045 ± 0.011 | -0.037 ± 0.010 | -0.037 ± 0.008 | | -0.037 ± 0.011 |
| **DN** | 0.030 ± 0.020 | 0.032 ± 0.017 | 0.038 ± 0.020 | | 0.032 ± 0.011 |
| **Br** | -0.049 ± 0.010 | -0.038 ± 0.011 | | -0.035 ± 0.011 | -0.039 ± 0.009 |
|  |  | | | | |
|  | **Volume (a.u.)** | | | | |
|  | **HC** | **DM1** | | **E1** | **E2** |
| **Cau** | 0.211 ± 0.016 | 0.213 ± 0.016 | 0.207 ± 0.010 | | 0.228 ± 0.018 |
| **Acc** | 0.022 ± 0.004 | 0.021 ± 0.002 | 0.020 ± 0.004 | | 0.021 ± 0.002 |
| **Put** | 0.242 ± 0.015 | 0.236 ± 0.017 | 0.238 ± 0.018 | | 0.235 ± 0.017 |
| **GP** | 0.082 ± 0.005 | 0.088 ± 0.006 | 0.088 ± 0.010 | | 0.088 ± 0.005 |
| **SN** | 0.030 ± 0.002 | 0.031 ± 0.002 | 0.031 ± 0.003 | | 0.031 ± 0.002 |
| **RN** | 0.013 ± 0.001 | 0.013 ± 0.001 | 0.013 ± 0.001 | | 0.013 ± 0.001 |
| **Th** | 0.488 ± 0.022 | 0.490 ± 0.023 | 0.497 ± 0.027 | | 0.487 ± 0.025 |
| **Hipp** | 0.247 ± 0.015 | 0.248 ± 0.019 | 0.267 ± 0.019 | | 0.245 ± 0.017 |
| **Amy** | 0.077 ± 0.013 | 0.085 ± 0.008 | 0.088 ± 0.008 | | 0.083 ± 0.009 |
| **DN** | 0.030 ± 0.003 | 0.032 ± 0.003 | 0.032 ± 0.002 | | 0.033 ± 0.004 |
| **Br** | 2.282 ± 0.102 | 2.325 ± 0.207 | 2.244 ± 0.220 | | 2.378 ± 0.165 |

Median χ (top) and volume (bottom) values (median ± mean absolute deviation) in HC, DM1, E1 and E2 groups in the analyzed subcortical structures. Median χ is expressed in parts per million (ppm). Volumes were corrected by total intracranial volume using the proportional method, so they are in arbitrary units (a.u.). (Cau = Caudate; Acc = Accumbens; Put = Putamen; GP = Globus Pallidus; SN = Substantia Nigra; RN = Red Nucleus; Th = Thalamus; Hipp = Hippocampus; Amy = Amygdala; DN = Dentate Nucleus; Br = Brainstem)

**Supplementary Figure S3:**


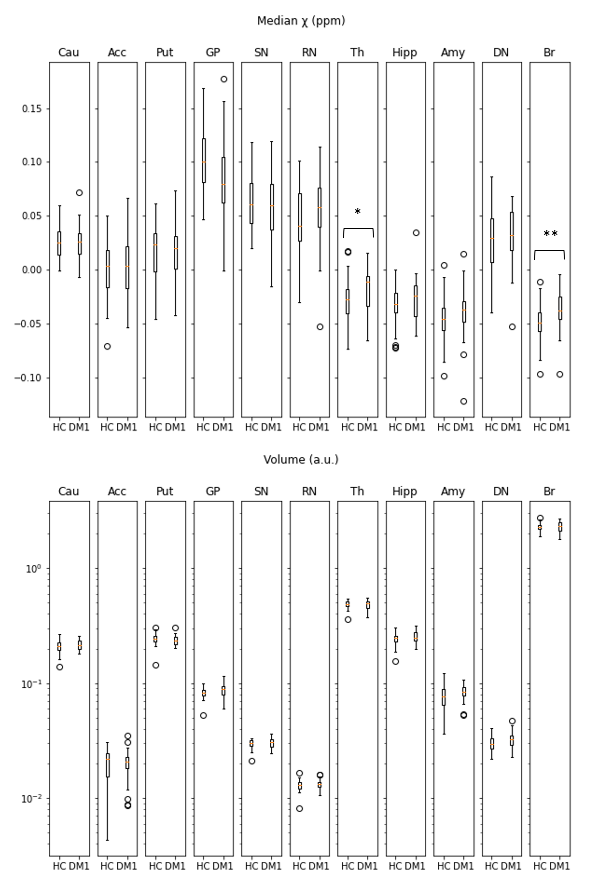


A

B

Bar plots of χ (A) and volume (B) distributions of the analyzed subcortical structures.

Median χ is expressed in parts per million (ppm). Volumes were corrected by total intracranial volume using the proportional method, so they are in arbitrary units (a.u.). VOI-based analysis was performed (Kruskal-Wallis test, * *p* < .05, ** *p* < .01) (Cau = Caudate; Acc = Accumbens; Put = Putamen; GP = Globus Pallidus; SN = Substantia Nigra; RN = Red Nucleus; Th= Thalamus; Hipp = Hippocampus; Amy = Amygdala; DN = Dentate Nucleus; Br = Brainstem)

**Supplementary Table S3:**

|  |  | **Median χ (ppm)** | | | | |
| --- | --- | --- | --- | --- | --- | --- |
|  |  | **HC** | **DM1** | | **E1** | **E2** |
| **Thalamus** | **Anterior** | -0.033 ± 0.010 | -0.030 ± 0.021 | -0.031 ± 0.024 | | -0.028 ± 0.017 |
|  | **Medial** | -0.039 ± 0.011 | -0.031 ± 0.018 | -0.032 ± 0.023 | | -0.029 ± 0.018 |
|  | **Ventral** | -0.037 ± 0.014 | -0.021 ± 0.013 | -0.021 ± 0.012 | | -0.024 ± 0.012 |
|  | **Pulvinar** | 0.008 ± 0.012 | 0.023 ± 0.019 | 0.020 ± 0.017 | | 0.029 ± 0.021 |
|  | **Total** | -0.027 ± 0.013 | -0.011 ± 0.017 | -0.018 ± 0.020 | | -0.011 ± 0.019 |
| **Brainstem** | **Midbrain** | -0.029 ± 0.010 | -0.021 ± 0.014 | -0.017 ± 0.009 | | -0.027 ± 0.011 |
|  | **Pons** | -0.054 ± 0.010 | -0.046 ± 0.011 | -0.043 ± 0.013 | | -0.046 ± 0.008 |
|  | **Medulla** | -0.042 ± 0.007 | -0.029 ± 0.012 | -0.030 ± 0.010 | | -0.029 ± 0.015 |
|  | **Total** | -0.049 ± 0.010 | -0.038 ± 0.011 | -0.035 ± 0.011 | | -0.039 ± 0.009 |

Median χ values (median ± mean absolute deviation) in HC, DM1, E1 and E2 groups in thalamic nuclei (anterior, medial, ventral and pulvinar) and brainstem sub-structures (midbrain, pons, medulla). Median χ is expressed in parts per million (ppm). ‘Total’ refers to the entire structure (thalamus and brainstem, respectively)

**Supplementary Table S4:**

|  |  | **Color** | **RGB** | **Brain area** |
| --- | --- | --- | --- | --- |
| **A** | Subcortical structures |  | 252,247,21 | Putamen Nucleus |
|  |  |  | 212,56,201 | Pallidus Nucleus |
|  |  |  | 221,173,204 | Thalamus |
|  |  |  | 233,35,195 | Nucleus Accumbens |
|  |  |  | 255,127,101 | Caudate Nucleus |
|  |  |  | 106,250,192 | Amygdala |
|  |  |  | 16,252,156 | Hippocampus |
| **B** | Thalamic nuclei |  | 91,74,194 | Medial Nuclei |
|  |  |  | 253,199,243 | Ventral Nuclei |
|  |  |  | 246,250,64 | Anterior Nuclei |
|  |  |  | 194,24,133 | Pulvinar |
| **C** | Brainstem structures |  | 58,176,248 | Midbrain |
|  |  |  | 189,195,251 | Medulla |
|  |  |  | 189,250,251 | Pons |
| **D** | RN, SN and DN |  | 209,227,41 | Red Nucleus |
|  |  |  | 36,144,49 | Substantia Nigra |
|  |  |  | 96,21,247 | Dentate Nucleus |

Reference

Brun G, Testud B, Girard OM, Lehmann P, de Rochefort L, Besson P, Massire A, Ridley B, Girard N, Guye M, Ranjeva JP, Le Troter A. Automatic segmentation of deep grey nuclei using a high-resolution 7T magnetic resonance imaging atlas-Quantification of T1 values in healthy volunteers. Eur J Neurosci. 2022 Jan;55(2):438-460. doi: 10.1111/ejn.15575. Epub 2022 Jan 7. PMID: 34939245.

**MR image processing**

In all the processing steps below, the subject’s images are masked using FSL bet so that only brain voxels are taken into consideration.

**Atlas construction**

**Thalamic nuclei.** The 7TAMIbrain atlas of Brun and colleagues^53^ was used to define 4 thalamic nuclei. It is not registered to MNI152 space.

**Subthalamic nuclei.** All the healthy control images in the study were registered to the standard MNI152 image volume in two stages. Each subject's QSM magnitude image (0.5x0.5x1 mm) was registered to his or her MPRAGE image volume (1x1x1 mm) using ANTs with default options. The result was registered to MNI152 space by rigid registration of the MPRAGE volume to the standard MNI152 T1 weighted image, using FSL tool flirt with options ‘-searchrx -90 90 -searchry -90 90 -searchrz -90 90 -noresampblur’. The average QSM magnitude map in MNI152 space was thus created and used for the manual definition of subthalamic nuclei masks as described in the Methods.

**Segmentation**

**FreeSurfer.** Segmentation of cortical and brainstem structures was performed using freesurfer v.6, with default settings apart from the use of the option ‘-brainstem-structures’.

**First.** The FSL utility First was run as follows:

run_first_all -d -m auto -i sub-01_T1w.nii.gz -s L_Accu,L_Amyg,L_Caud,L_Hipp,L_Pall,L_Puta,L_Thal,L_Late,R_Accu,R_Amyg,R_Caud,R_Hipp,R_Pall,R_Puta,R_Thal,R_Late -o *$output_directory*/sub-01/T1

**Intrasubject registration**

For each subject, the QSM magnitude image volume was linearly registered with the MPRAGE image volume using FSL tool flirt with default options.

**ROI definition**

**Cortical ROIs and deep grey matter ROIs.** These were available from the FreeSurfer and First segmentations in the subject’s own T1W space in which statistical calculations were performed.

**Thalamic nuclei ROIs.** Following the procedure recommended by Brun and colleagues, the 7TAMIbrain volume was registered to each subject’s T1-weighted brain image using ANTs, as follows:

antsRegistrationSyN.sh -d 3 -m 7TAMI_T1w_30.nii.gz -f sub-01_T1w.nii.gz -o 7TAMI_2_subject_SyN

The registration parameters were used to take the atlas to the subject’s native space:

antsApplyTransforms -d 3 -e 0 -i 7TAMI_DGN.nii.gz -r sub-01_T1w.nii.gz -o DGN_mask_subj.nii.gz -t 7TAMI_2_subject_SyN1Warp.nii.gz -t 7TAMI_2_subject_SyN0GenericAffine.mat -n NearestNeighbor

**Subthalamic nuclei ROIs.** The MPRAGE image volume was non-linearly registered to the standard MNI152 T1 weighted image in two stages, first using FSL flirt and then FSL fnirt

flirt -in sub-01_T1w.nii.gz -ref MNI152_brain -omat T1_to_MNI_lin_sub-01.mat -out T1_brain_MNI_lin_sub-01.nii.gz -searchrx -90 90 -searchry -90 90 -searchrz -90 90 -noresampblur

convert_xfm -omat MNI_to_T1_lin_sub-01.mat -inverse T1_to_MNI_lin_sub-01.mat

fnirt --ref=sub-01_T1w.nii.gz --in= MNI152_brain --aff=MNI_to_T1_lin_sub-01.mat --cout=MNI_to_T1_warp_sub-01.nii.gz

The inverse transformation was applied to take ROIs in MNI space to subject’s native space (for example, for left substantia nigra):

applywarp --ref=sub-01_T1w.nii.gz --in=ROI_L-SN --warp= MNI_to_T1_lin_sub-01.nii.gz --out= MNI_to_T1_lin_sub-01.nii.gz --interp=nn
